# E2f5 is a versatile transcriptional activator required for spermatogenesis and multiciliated cell differentiation in zebrafish

**DOI:** 10.1371/journal.pgen.1008655

**Published:** 2020-03-20

**Authors:** Haibo Xie, Yunsi Kang, Shuo Wang, Pengfei Zheng, Zhe Chen, Sudipto Roy, Chengtian Zhao

**Affiliations:** 1 Institute of Evolution & Marine Biodiversity, Ocean University of China, Qingdao, Shandong, China; 2 Laboratory for Marine Biology and Biotechnology, Qingdao National Laboratory for Marine Science and Technology, Qingdao, Shandong, China; 3 Ministry of Education Key Laboratory of Marine Genetics and Breeding, College of Marine Life Sciences, Ocean University of China, Qingdao, Shandong, China; 4 Institute of Molecular and Cell Biology, Singapore, Singapore; 5 Department of Biological Sciences, National University of Singapore, Singapore, Singapore; 6 Department of Pediatrics, Yong Loo Ling School of Medicine, National University of Singapore, Singapore, Singapore; Monash University, AUSTRALIA

## Abstract

E2f5 is a member of the E2f family of transcription factors that play essential roles during many cellular processes. E2f5 was initially characterized as a transcriptional repressor in cell proliferation studies through its interaction with the Retinoblastoma (Rb) protein for inhibition of target gene transcription. However, the precise roles of E2f5 during embryonic and post-embryonic development remain incompletely investigated. Here, we report that zebrafish E2f5 plays critical roles during spermatogenesis and multiciliated cell (MCC) differentiation. Zebrafish *e2f5* mutants develop exclusively as infertile males. In the mutants, spermatogenesis is arrested at the zygotene stage due to homologous recombination (HR) defects, which finally leads to germ cell apoptosis. Inhibition of cell apoptosis in *e2f5;tp53* double mutants rescued ovarian development, although oocytes generated from the double mutants were still abnormal, characterized by aberrant distribution of nucleoli. Using transcriptome analysis, we identified *dmc1*, which encodes an essential meiotic recombination protein, as the major target gene of E2f5 during spermatogenesis. E2f5 can bind to the promoter of *dmc1* to promote HR, and overexpression of *dmc1* significantly increased the fertilization rate of *e2f5* mutant males. Besides gametogenesis defects, *e2f5* mutants failed to develop MCCs in the nose and pronephric ducts during early embryonic stages, but these cells recovered later due to redundancy with E2f4. Moreover, we demonstrate that ion transporting principal cells in the pronephric ducts, which remain intercalated with the MCCs, do not contain motile cilia in wild-type embryos, while they generate single motile cilia in the absence of E2f5 activity. In line with this, we further show that E2f5 activates the Notch pathway gene *jagged2b* (*jag2b*) to inhibit the acquisition of MCC fate as well as motile cilia differentiation by the neighboring principal cells. Taken together, our data suggest that E2f5 can function as a versatile transcriptional activator and identify novel roles of the protein in spermatogenesis as well as MCC differentiation during zebrafish development.

## Author summary

E2f family of transcription factors play essential roles during many cellular processes by activating or inhibiting target gene expression. E2F5 is recognized as a transcriptional repressor during cell-cycle progression. However, the role of E2f5 during embryonic development has not been fully investigated. Here, we show that E2f5 is required both for spermatogenesis and multiciliated cell differentiation in zebrafish. Specifically, E2f5 regulates homologous recombination, a central event in meiosis, by transactivating the expression of *dmc1*. Loss of E2f5 leads to spermatogenesis defects due to meiosis arrest. In addition, E2f5 plays an essential role during the differentiation of multiciliated cells and ion transporting principal cells in the pronephric ducts (embryonic zebrafish kidney). We showed that principal cells were devoid of cilia in the proximal straight tubule of pronephric ducts, while ectopic cilia developed in these cells due to lateral inhibition defects in the absence of E2f5 protein. Our data suggest that E2f5 functions as a versatile transcriptional activator during vertebrate development.

## Introduction

E2f transcription factor family members are essential for cell proliferation, differentiation, apoptosis as well as many other cellular processes [1]. The roles of E2f proteins in cell-cycle control have been studied in great detail. In this context, interaction between Rb and E2f family members inhibits the transcription of E2f target genes at the G1 phase. Subsequently, phosphorylation of Rb by cyclin/cyclin-dependent kinases releases E2fs, which allows activation of their target genes and promotes G1 to S transition [2]. Consequently, unrestrained E2f activity due to inactivation of Rb, occurs in many types of cancers [2, 3].

Currently, eight different E2f proteins (E2f1-E2f8) have been identified, which can be generally classified into three categories according to their roles during cell cycle progression: transcription activators (E2f1, E2f2 and E2f3a), repressors (E2f3b, E2f4 and E2f5) and inhibitors (E2f6, E2f7 and E2f8) [4]. E2f1-3 are categorized as classic activators due to their transcriptional activity when binding to the promoter of target genes during cell-cycle entry. E2f4 and E2f5 function as transcription repressors through interactions with pocket proteins p107, p130, and Rb to inhibit target gene transcription during early G1 phase [4, 5]. Nevertheless, such classification is largely based on *in vitro* experiments probing their roles during the cell-cycle, which could be oversimplified, and may not necessarily reflect the complicated activities of E2f proteins *in vivo* [2].

Although being recognized as transcriptional repressors during cell cycle regulation, our current appreciation of the roles of E2f4 and E2f5 during embryonic development suggests alternative mechanisms of function. Mouse *E2f4*^*-/-*^ mutants display defects in multiple tissues, and most die within the first week after birth due to increased susceptibility to opportunistic infections resulting from defects in the generation of MCCs within the airways [6–8]. E2f4 is also required for the development of MCCs within the male reproductive system as conditional knockout of *E2f4* in *E2f5*^*+/-*^ mice caused defects in the efferent ducts of the testes, where sperm are concentrated and transported into the epididymis [9]. By contrast, the role of E2f5 during embryonic development is relatively less well investigated. *E2f5*^*-/-*^ mice have a shortened lifespan and develop hydrocephalus due to excessive cerebrospinal fluid (CSF) production [10]. Interestingly, mouse embryonic fibroblasts (MEFs) derived from both *E2f4*^*-/-*^ and *E2f5*^*-/-*^ mice display normal cell-cycle kinetics [6, 10]. All of these data suggest that the role of E2f4 and E2f5 during embryonic development is context-dependent, as opposed to these proteins functioning as general repressors in cell-cycle regulation.

MCCs are a specialized type of post-mitotic cells characterized by differentiation of hundreds of motile cilia that can beat unidirectionally to drive fluid flow over their epithelial surface. In mammals, MCCs are present in multiple organs, such as the spinal cord and brain ventricles, where they drive the flow of cerebrospinal fluid; the airways epithelia, where they help clearance of mucus; and in the fallopian tubes, where they are involved in ovum transport [11, 12]. The Notch signaling pathway is known to play a critical role during determination of the MCC fate [11]. Inhibition of Notch signaling leads to an expansion of MCC numbers at the expense of other cell types [13–17] in various tissues where this phenomenon has been examined. For example, in the zebrafish pronephric duct, the Notch ligand Jag2b is expressed in developing MCCs. Interaction between Jag2b and Notch1a/Notch3 receptors, a process called lateral inhibition, orchestrates the segregation of the “salt-and-pepper” pattern of MCCs and principal cells in the pronephros [13, 18].

Geminin coiled-coil domain-containing protein 1 (Gemc1, aka Gmnc) and Multicilin (Mci, aka Mcidas) are the known upstream activators of MCC differentiation downstream of Notch signals [19–22]. Interestingly, both proteins are devoid of an obvious DNA binding domain, although they both localize to the nucleus. Instead, Gemc1 and Mci associate with E2f4 and/or E2f5, together with Dp1 to activate gene expression for MCC specification and differentiation [20, 21, 23, 24]. Intriguingly, besides functioning as a transcription regulator, cytoplasmic E2f4 has also been shown to participate in the assembly of deuterosomes in the MCCs—electron-dense ring-like structures that are thought to seed the massive amplification of centrioles for multiciliation [25, 26].

Homologous recombination (HR) is an essential step during meiosis when homologous chromosomes pair and undergo reciprocal exchange of DNA during the first meiotic division. After homologous chromosome pairing, meiotic recombination is initiated by the formation of double-strand breaks (DSBs) at multiple positions through the actions of several factors including the topoisomerase-like enzyme Spo11. Later, binding of Dmc1 and Rad51 to the DSBs generates nucleoprotein filaments and initiates proper strand invasion to form D-loops. The DSBs are repaired either through a reciprocal exchange of chromosome arms by forming a crossover or non-crossover where no chromosome exchange happens [27, 28]. Both Rad51 and Dmc1 are recombinases that are required for strand invasion during meiotic HR, but they have overlapping and distinct functions. Rad51 regulates HR both in somatic cells and germ cells, whereas Dmc1 is expressed only in meiotic cells [29]. Several pieces of evidence suggest that Rad51 mainly performs an accessory function to promote Dmc1 foci formation during meiosis [30, 31]. Currently, the role of E2f5 during spermatogenesis, especially in meiosis, has not been reported.

We have generated loss-of-function alleles in zebrafish *e2f5* and investigated the role of the gene during embryogenesis and adult development. Zebrafish *e2f5* mutants display sex differentiation defects and develop exclusively as males. Interestingly, all male mutants are infertile due to meiotic arrest of spermatocytes. We have identified Dmc1 as the major factor accounting for HR defects in the mutants, and further show that E2f5 can bind to the promoter of *dmc1*, but not *rad51*. Overexpression of Dmc1 rescued spermatogenesis defects in *e2f5* mutants. In addition, we found that E2f5 is not only required for MCC formation, but is also required cell-nonautonomously to orchestrate the differentiation of neighboring ion transporting principal cells by regulating the expression of *jag2b* in MCC precursors.

## Results

### Mutation of *e2f5* results in male infertility in zebrafish

**Fig 1:**
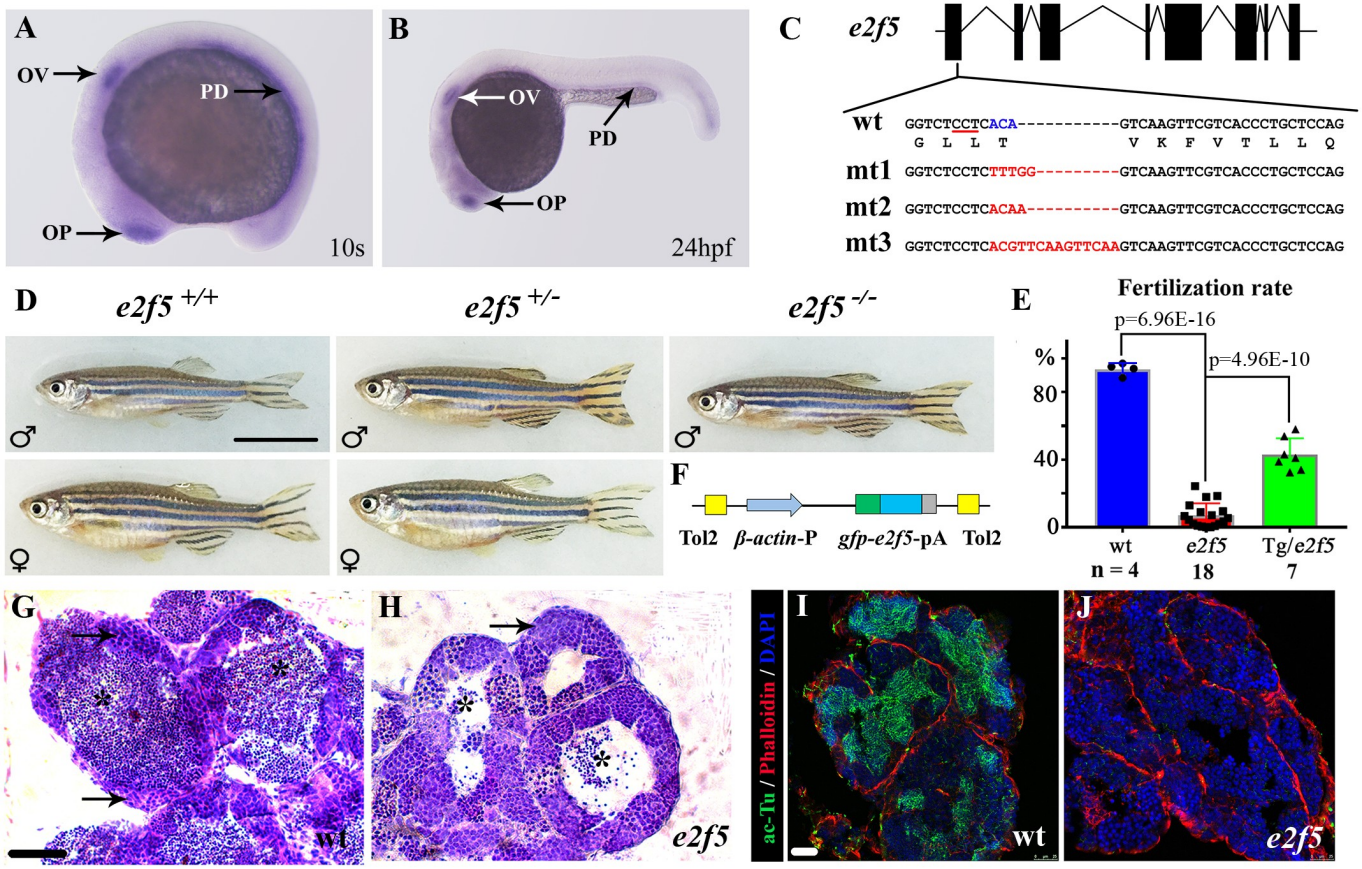
Mutation of *e2f5* leads to male infertility. (A-B) Whole-mount *in situ* hybridization showing expression of *e2f5* at 10-somite stage (10s) and 24 hpf. OP, olfactory placode; OV, otic vesicle; PD, pronephric duct. (C) Genomic structure and sequences of wild- type (wt) and three *e2f5* mutant alleles. The underlined sequence in wt indicates PAM sequence of sgRNA target. (D) Phenotypes of male and female wild-type and *e2f5* heterozygotes. Only fish exhibiting the male phenotype were present among homozygous mutants. (E) Bar graph showing the percentage of fertilization rates of wild-type, *e2f5* homozygote and *e2f5* homozygotes carrying *Tg(β-actin*:*gfp-e2f5)* transgene as indicated. The numbers of adult males investigated are listed at the bottom. (F) Diagram of the constructs for making *gfp*-*e2f5* transgene. (G-H) H&E staining results showing testes of wild-type (G) and *e2f5* homozygous mutants (H). Arrows indicate primary spermatocytes. Asterisks point to mature spermatozoa which were substantially reduced in the mutants. (I-J) Confocal images showing the staining of sperm flagella in wild-type (I) and *e2f5* mutant testis (J) visualized with acetylated alpha-tubulin (ac-Tu) antibody. Nuclei and actin filaments were counterstained with DAPI and phalloidin, respectively. Scale bars: 1cm in panel D, 50 μm in panel G, H and 25 μm in panel I, J.

We first analyzed the expression pattern of *e2f5* using whole-mount *in situ* hybridization. *e2f5* showed tissue-specific expression at early developmental stages, with high levels of expression in the olfactory placode, otic vesicle and pronephric ducts at the 10-somite stage (Fig 1A) and 24 hours post-fertilization (hpf) (Fig 1B). At 24hpf, *e2f5* was also strongly expressed in the posterior spinal cord (Fig 1B).

To elucidate the function of E2f5, we generated three zebrafish *e2f5* alleles, all of which contained frameshift mutations predicted to cause premature termination during translation and a complete loss-of-function condition (Fig 1C). Surprisingly, animals homozygous for all three mutant alleles are viable. However, all homozygous *e2f5* mutants develop exclusively as males. Of a total of 723 adults from crosses between heterozygote mutants, we identified 245 wild-type, 330 heterozygote and 148 homozygous mutants, implying a slightly decreased survival rate of homozygous mutants. While both wild-type and heterozygote fish displayed a similar ratio between male and female, none of the 148 homozygous mutants were female (Fig 1D). When further crossed to wild-type females, all of the homozygous mutants from all three mutant alleles displayed severe fertility defects (the percentage of fertilization rates in mutants ca. 3%, n = 18; vs wild-type 94%, n = 4) (Fig 1E). To further explore this phenotype, we generated a stable transgenic line of E2f5 fused to the C-terminus of GFP, expression of which is driven by the constitutive and ubiquitously active *β-actin* promoter (Fig 1F). The fertilization rate was significantly rescued in *e2f5* mutant males carrying this transgene (Fig 1E). Moreover, this transgene also allowed the recovery of female *e2f5* homozygous mutants (S1A and S1B Fig). These data suggest that the fertility defects are due to a *bona fide* requirement of E2f5 in the germline.

In adult wild-type males, fully developed testes are plump and display milky white color, while testes from *e2f5* mutants are slender and the color is also quite transparent (S1C and S1D Fig). Furthermore, histological sections through the seminiferous tubules showed a large number of mature spermatozoa and spermatocytes in the wild-type, while the number of mature spermatozoa were reduced substantially in the mutant testes (Fig 1G and 1H). Immunostaining with acetylated tubulin antibody, which labels the flagella of mature spermatozoa, also showed the absence of mature spermatozoa in the mutant testes (Fig 1I and 1J). These results demonstrate that spermatogenesis is strongly impaired in the absence of E2f5 activity.

### Spermatogenesis is arrested in prophase I of meiosis in *e2f5* mutants

**Fig 2:**
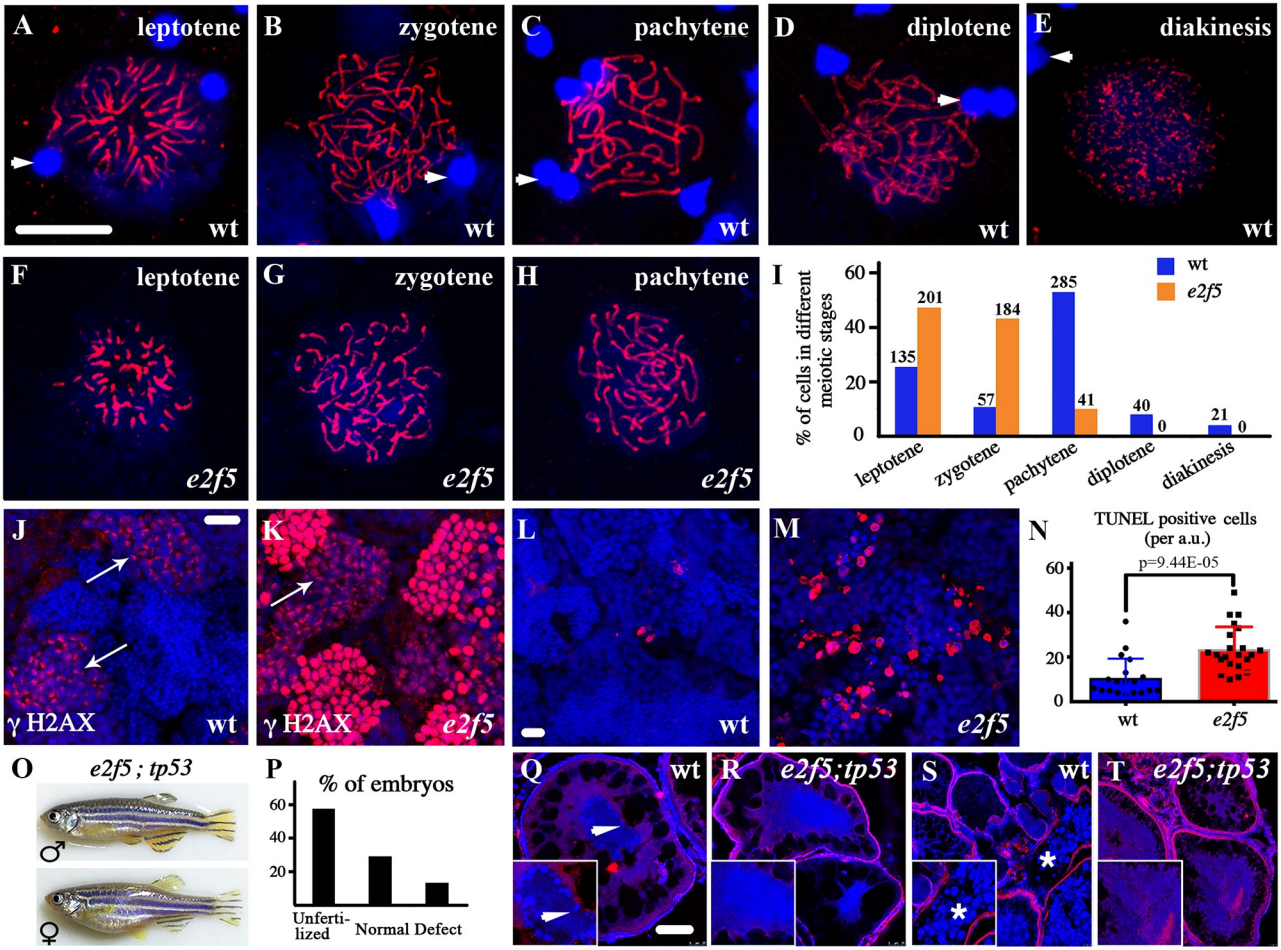
Spermatogenesis arrest in *e2f5* mutants. (A-H) Confocal images of primary spermatocytes at different prophase stages of meiosis I as indicated by anti-Sycp3 antibody staining (red). Arrowheads indicate spermatozoa nuclei from wild-type testis stained with DAPI in blue (A-E). (I) Bar graph showing the statistical results of the percentage of cells in different meiotic stages. The numbers of spermatocytes investigated are shown on top of each bar. (J-K) Staining of γH2AX (red) in the testes of wild-type and *e2f5* mutant. Arrows point to the primary spermatocytes at leptotene and zygotene stages. (L-M) Confocal images showing apoptotic cells stained by TUNEL assay in red. (N) Bar graph with individual data points showing the number of TUNEL positive cells in wild-type and *e2f5* mutant testes. (O) Phenotypes of *e2f5;tp53* double mutants. (P) Bar graph showing the percentage of abnormal embryos produced from *e2f5;tp53* double mutant females crossed with wild-type males. n = 441 from two double mutant females. (Q-T) Histological analysis of ovaries from wild-type and *e2f5;tp53* double mutants. Inserted images are magnified views. Arrow in Q indicates peripheral localization of nucleoli stained by DAPI in stage II oocytes of wild-type ovary. The asterisk in S indicates cortical alveoli at later stage oocytes, which were visible under the confocal microscope due to autofluorescence. In panels J-M, Q-T, nuclei were counterstained with DAPI in blue. Scale bars: 10 μm in panel A-H, J-M and 50 μm in panel Q-T.

After DNA replication and sister chromatid formation is complete, primary spermatocytes enter the first meiotic division (prophase I). Absence of mature spermatozoa is suggestive of defective meiosis in *e2f5* mutants. To begin to understand the defect in greater detail, we analyzed the expression of Synaptonemal complex protein 3 (Sycp3), a marker of meiotic prophase I. Sycp3 is detected first in the lateral element of the synaptonemal complex at leptotene, and remains as part of this complex during homologous synapsis at zygotene and pachytene stages. At the later diplotene and diakinesis stages, Sycp3 signals become weakened and is maintained as short patches between the sister chromatids (Fig 2A–2E) [32]. In *e2f5* mutants, we observed similar distribution of Sycp3 at early stages (Fig 2F–2H). However, the percentage of spermatocytes at leptotene and zygotene stages were increased dramatically, while those at later stages were barely seen (Fig 2I), confirming a meiotic arrest of spermatogenesis.

Phosphorylation of H2A histones is indispensable for the recruitment of repair factors to damaged DNA after DSB formation in leptotene and zygotene spermatocytes. In the wild-type, staining of phosphorylated histone H2AX (γ-H2AX) first appeared as clusters in the nuclear region at mid-leptotene stage, and then displayed a scattered pattern throughout the nucleus at late leptotene and early zygotene stages (Fig 2J). From mid-zygotene stage, γ-H2AX staining gradually disappeared and no staining was detected from late zygotene onward [33, 34]. In contrast, high levels of γ-H2AX staining was present in the mutant testes. In addition to the clusters of staining at mid-leptotene stage, most cells had strong γ-H2AX staining throughout their nuclei (Fig 2K), suggesting that spermatogenesis likely arrests at the zygotene stage in the absence of E2f5.

### Apoptosis in *e2f5* mutant spermatocytes

Germ cell apoptosis is a common consequence of meiotic arrest during spermatogenesis. The number of apoptotic cells increased significantly in the testes of *e2f5* mutants (Fig 2L–2N). It has been shown earlier that mutation of the apoptotic gene *tp53* (*p53*) can rescue the sex reversal defects caused by germ cell apoptosis [35, 36]. To test this, we generated *e2f5*^*-/-*^; *tp53*^*-/-*^ double mutants to prevent cell apoptosis. Although double mutant males were still infertile, *e2f5* female homozygous mutants could be recovered in the absence of p53 activity (4 female and 4 male double mutants were recovered among 128 adult fish derived from crossing between two *e2f5*^*+/-*^; *tp53*^*+/-*^ heterozygotes) (Fig 2O), suggesting that p53 mediated germ cell apoptosis contributes to the absence of female *e2f5* mutants. Interestingly, *e2f5*^*-/-*^; *tp53*^*-/-*^ females were able to produce eggs when crossed to wild-type males, although the fertilization rates were low and most fertilized eggs developed abnormally at later stages (Fig 2P, S1E and S1F Fig). Further histological analysis showed that the symmetrical distribution of nucleoli near the nuclear periphery (stage IB oocyte) was consistently disrupted in oocytes from the double mutants (Fig 2Q and 2R). In addition, late-stage oocytes, normally distinguished by numerous cortical alveoli, were also barely seen in the mutant ovaries (Fig 2S and 2T). Thus, even though ovaries formed, oocyte development remained defective in the double mutant females.

### Transcriptome analysis of *e2f5* mutants

**Fig 3:**
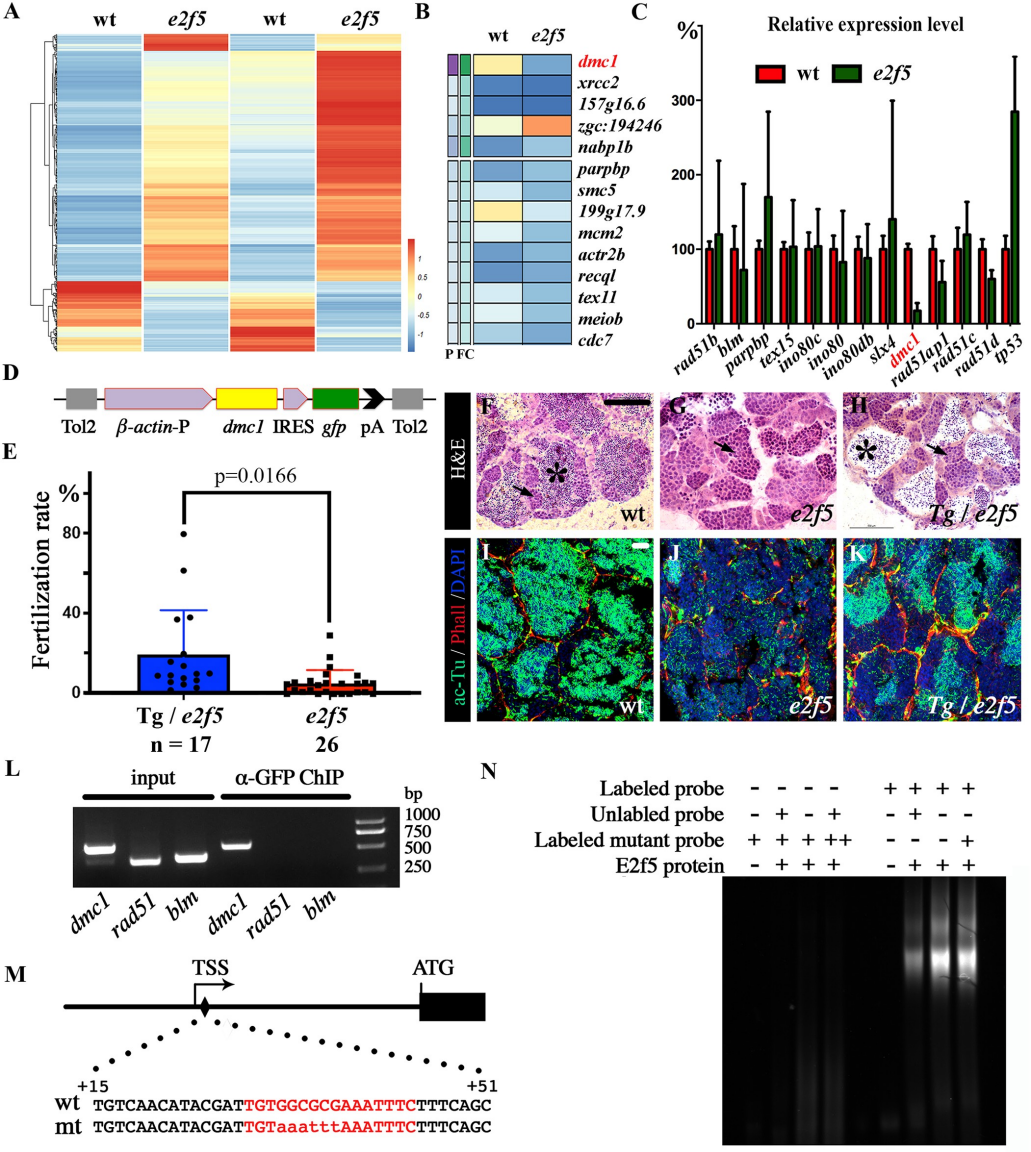
E2f5 binds to the promoter of *dmc1*. (A) Heat map of RNA-seq transcriptome analysis from two sets of wild-type and mutant testes. (B) Expression heat map of genes involved in homologous recombination in wild-type and *e2f5* mutants. The full list of genes analyzed is listed in S2A Fig. (C) qRT-PCR results showing the relative expression level of genes involved in homologous recombination in wild-type and *e2f5* mutant testes. The expression of each gene in wild-type testes was set as 100%. The full list of genes analyzed is listed in S2B Fig. (D) Diagram of the construct used for generating *Tg(β-actin*:*dmc1)* transgenic fish. (E) Dot plot showing the fertilization rate of *e2f5* mutants carrying the *Tg(β-actin*:*dmc1)* transgene. (F-H) H&E staining results showing testes from wild-type, *e2f5* mutant and *e2f5* mutant carrying *Tg(β-actin*:*dmc1)* transgene. Asterisks indicate mature spermatozoa. Arrows indicate spermatocytes. (I-K) Confocal images showing the staining of sperm flagella visualized with an anti-acetylated tubulin antibody in green in wild-type and mutant testes as indicated. Nuclei and actin filaments were counterstained with DAPI (blue) and phalloidin (red) respectively. (L) CHIP analysis of GFP-E2f5 binding to the promoter of *dmc1*. (M) Diagram showing the position and sequence of potential E2f5 binding site near the transcription start site (TSS) of the *dmc1* gene. (N) EMSA analysis of the interaction between E2f5 protein and oligonucleotide probes corresponding to the wild-type and mutant E2F binding sites as indicated in panel M. “++” indicates that the amount of probes was doubled than those in other parallel experiments (+). Scale bars: 100 μm in panel F-H and 25 μm in panel I-K.

To further clarify the reason of meiotic arrest in *e2f5* mutants, we performed RNA sequencing (RNA-seq) analysis on the testes of wild-type and *e2f5* mutants. Sequencing results from two independent sample sets (in total from 10 wild-type and 10 mutants) revealed distinct gene expression patterns of *e2f5* mutant testes relative to those of wild-type controls (Fig 3A). We found 1352 transcripts upregulated in *e2f5* mutants compared to wild-type, whereas 763 transcripts were downregulated (FDR< = 0.05). Surprisingly, most genes associated with homologous recombination, including *rad51*, *blm*, *ino80* and *brca2* [37, 38], showed grossly normal expression levels in the mutants (Fig 3B, S2A Fig). However, the expression of *dmc1*, an essential gene involved in meiosis homologous recombination, was significantly downregulated in the mutants (Fig 3B, S2A Fig). To further validate this finding, we compared the expression levels of genes involved in meiotic HR via qRT-PCR. Of all the genes tested, *dmc1* was again the only one significantly down regulated in *e2f5* mutant testes (p = 4.21E-24), while other genes, including *rad51*, were expressed at similar levels to those of the wild-type controls (Fig 3C, S2B Fig). Expression level of *tp53* was also increased, which is consistent with the TUNEL assay results (Fig 3C).

### Overexpression of Dmc1 rescued spermatogenesis defects in *e2f5* mutants

Mutation of *DMC1* in humans and mice leads to male sterility due to defects in spermatogenesis [39]. In the medaka fish, loss-of-function of Dmc1 has been reported to produce abnormal sperm with multiple tails [40]. In line with this, we also observed abnormal sperm with two flagella in zebrafish *e2f5* mutants (S3 Fig), suggesting that the decreased expression of Dmc1 could be the key issue underlying the defects in spermatogenesis in *e2f5* mutants. To test this hypothesis directly, we generated a stable transgenic line expressing *dmc1* under the control of the *β-actin* promoter (Fig 3D). With this transgene, the fertilization rates were significantly increased in *e2f5* homozygous mutants (Fig 3E). The majority of *e2f5* homozygous mutants (19 of 26 males) have fertilization rates lower than 5%, while 9 of 17 *e2f5* mutants containing *dmc1* transgene showed fertilization rates above 10%. Of note, in some of the transgenic lines, the fertilization rates increased to 77%, comparable to the rescue efficiency observed in Tg(*β-actin*:*gfp-e2f5*) transgenic *e2f5* mutants (Figs 3E vs 1E). Histological analysis and immunostaining with acetylated tubulin antibody revealed that the number of mature spermatozoa was also substantially increased in the testes of the transgenic mutants (Fig 3F–3K).

### E2f5 binds to the promoter of *dmc1*

To investigate whether E2f5 can regulate *dmc1* gene expression directly, we performed chromatin immunoprecipitation (CHIP) analysis. We dissected testes from Tg(*β-actin*:*gfp-e2f5*) transgenic fish and incubated the lysate with anti-GFP antibody to pull down E2f5-binding DNA fragments. Following this, PCR analysis showed a –309 to +245 DNA fragment, near the transcription start site of *dmc1*, to be significantly enriched for E2f5-binding (Fig 3L). On the contrary, we failed to amplify any transcription elements of *rad51* and *blm* genes, which are both involved in HR, but show normal expression levels in *e2f5* mutants (S2A Fig). To confirm the binding activity of E2f5, we further performed electrophoretic mobility shift assays (EMSAs). By searching the promoter of *dmc1*, we identified one potential E2f5 binding site within the +29 to +44 region (Fig 3M). When incubated with recombinant zebrafish E2f5 protein, only FAM-labeled wild-type, but not mutant probe, showed band shift (Fig 3N). Together, these data suggest that E2f5 can transactivate the expression of *dmc1* by directly binding to its promoter region.

### Multiciliogenesis defects in *e2f5* mutants

**Fig 4:**
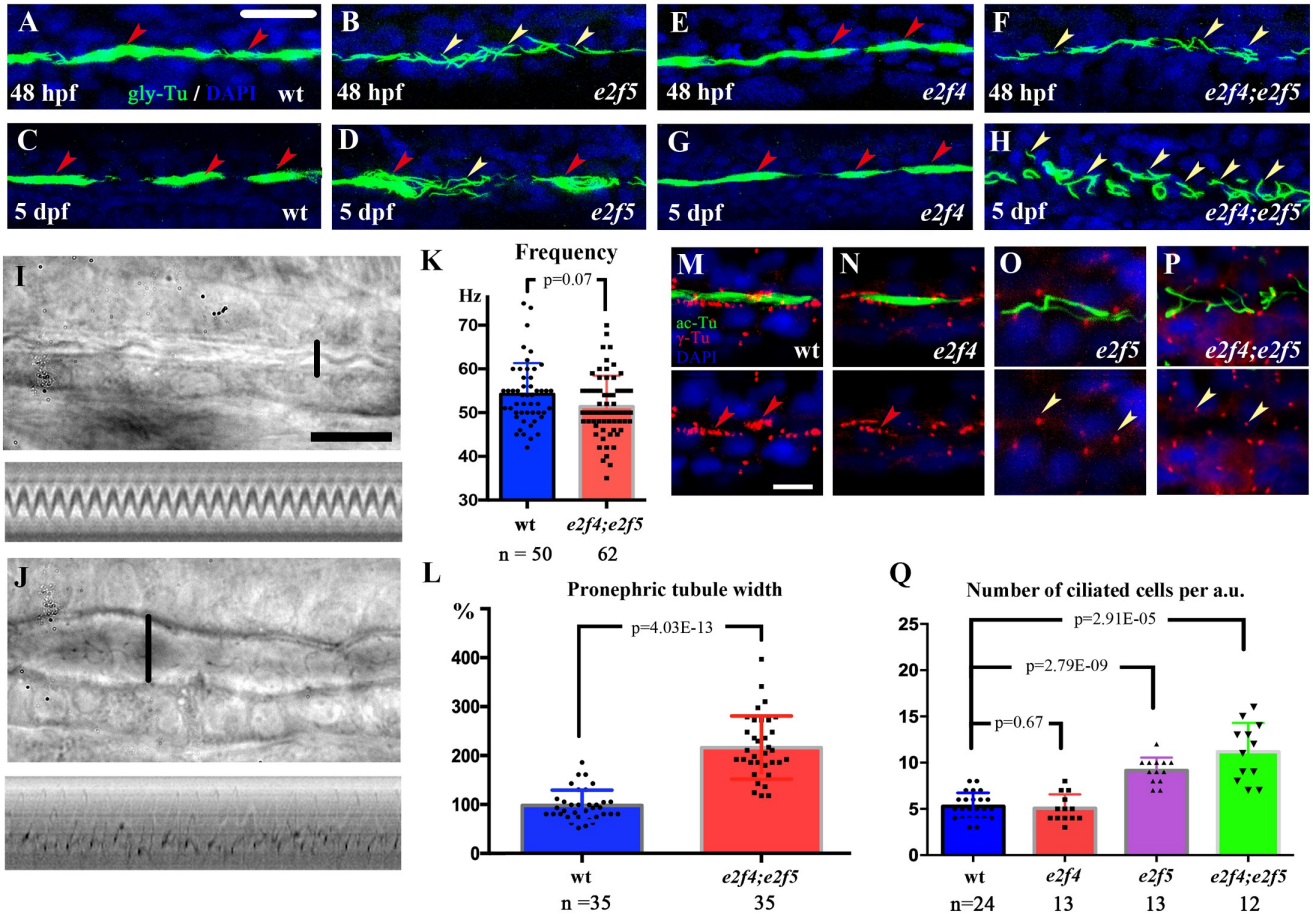
Multiciliogenesis defects in *e2f5* mutants. (A-H) Confocal images showing cilia in the PST region of the pronephros of wild-type and mutant larvae at different stages as indicated. Cilia were visualized with anti-glycylated tubulin antibody in green. Nuclei were labeled with DAPI in blue. Red arrowheads indicate multicilia bundles and yellow arrowheads indicate single cilia. (I-J) Still images from S1 and S2 Movies showing cilia in the PST of 5dpf wild-type (I) and *e2f4;e2f5* (J) double mutants. Bottom images show kymographs of cilia movement. (K) Beating frequency of cilia in the pronephric tubules of wild-type and *e2f4; e2f5* double mutants. (L) Dot plot showing the width of pronephric tubule lumen as indicated by vertical lines in panel I and J. (M-P) Confocal images showing cilia and basal bodies labeled with anti-acetylated tubulin (ac-Tu, green) and anti-γ tubulin (γ-Tu, red) antibodies in the PST of 48 hpf wild-type and mutant larvae as indicated. Staining of γ-tubulin is also indicated in the bottom to show the multiple basal bodies of MCCs (red arrowheads) and single ciliary basal bodies (yellow arrowheads). (Q) The number of cells bearing motile cilia per arbitrary unit (a.u.) in the PST region of wild-type and mutant larvae as indicated. Scale bars: 25 μm in panels A-H, 10 μm in panels I, J and 5μm in panels M-P.

E2f5 plays an essential role during multicilia formation in MCCs by regulating the expression of genes required for centriole duplication and ciliary motility [23, 24]. Moreover, gene ontology analysis of biological functions of the transcriptome in *e2f5* mutants also pointed to dramatic expression changes of genes required for ciliogenesis (S4 Fig). Whole-mount immunostaining results with anti-glycylated tubulin antibody showed that MCCs failed to develop in the mutant pronephric tubules and olfactory pits, while single cilia in spinal cord and ear hair cells developed normally (Fig 4A and 4B, S5A–S5H Fig). MCCs are enriched in the proximal tubule of zebrafish pronephros, which can be further subdivided into proximal convoluted tubule (PCT) and proximal straight tubule (PST) [41]. Interestingly, multicilia in the PST of pronephric tubules, but not PCT, recovered at 5dpf in the mutants (Fig 4C and 4D, S5G and S5H Fig). This could be due to the redundancy of function with E2f4. We therefore generated *e2f4* mutants to address this issue (S5I Fig). Although MCCs developed completely normally in the *e2f4* mutants, *e2f4;e2f5* double mutants displayed a complete block in multiciliogeneis both in the pronephric tubules and the nose (Fig 4E–4H, S5J–S5M Fig). On the other hand, the recovery of MCCs in the PST was completely inhibited in the double mutants and only single cilia were present at 5dpf (Fig 4H).

To gauge whether the single cilia of the double mutants are motile or immotile, we assessed cilia motility in the pronephric tubules with high-speed video microscopy. In the PST of 5 dpf wild-type larvae, coordinated cilia beating formed a rhythmic sinusoidal wave, a characteristic pattern of multicilia motility (Fig 4I, S1 Movie) [42]. By contrast, in *e2f4; e2f5* mutants, cilia movement appeared to be uncoordinated (Fig 4J, S2 Movie). Although ciliary beat frequency was similar to that of multicilia in wild-type embryos, the pronephric tubules were significantly dilated (Fig 4K–4L). Noticeably, all the cilia in the PST were single motile cilia, and the number of cells bearing cilia appeared to be increased in the mutants (S2 Movie). To further examine this, we labeled cilia and basal bodies with anti-acetylated alpha-tubulin and gamma-tubulin antibodies, respectively. In wild-type and *e2f4* mutant larvae, each multicilia bundle arose from numerous basal bodies (Fig 4M and 4N). In contrast, each cilium was associated with a single basal body in *e2f5* and *e2f4;e2f5* double mutants (Fig 4O and 4P). We compared the number of cells bearing cilia in the PST region and found the number of ciliated cells increased significantly (Fig 4Q). Together, these data suggest that differentiation of the MCCs is interrupted in *e2f4; e2f5* double mutants.

### Cilia are not present in the PST principal cells

**Fig 5:**
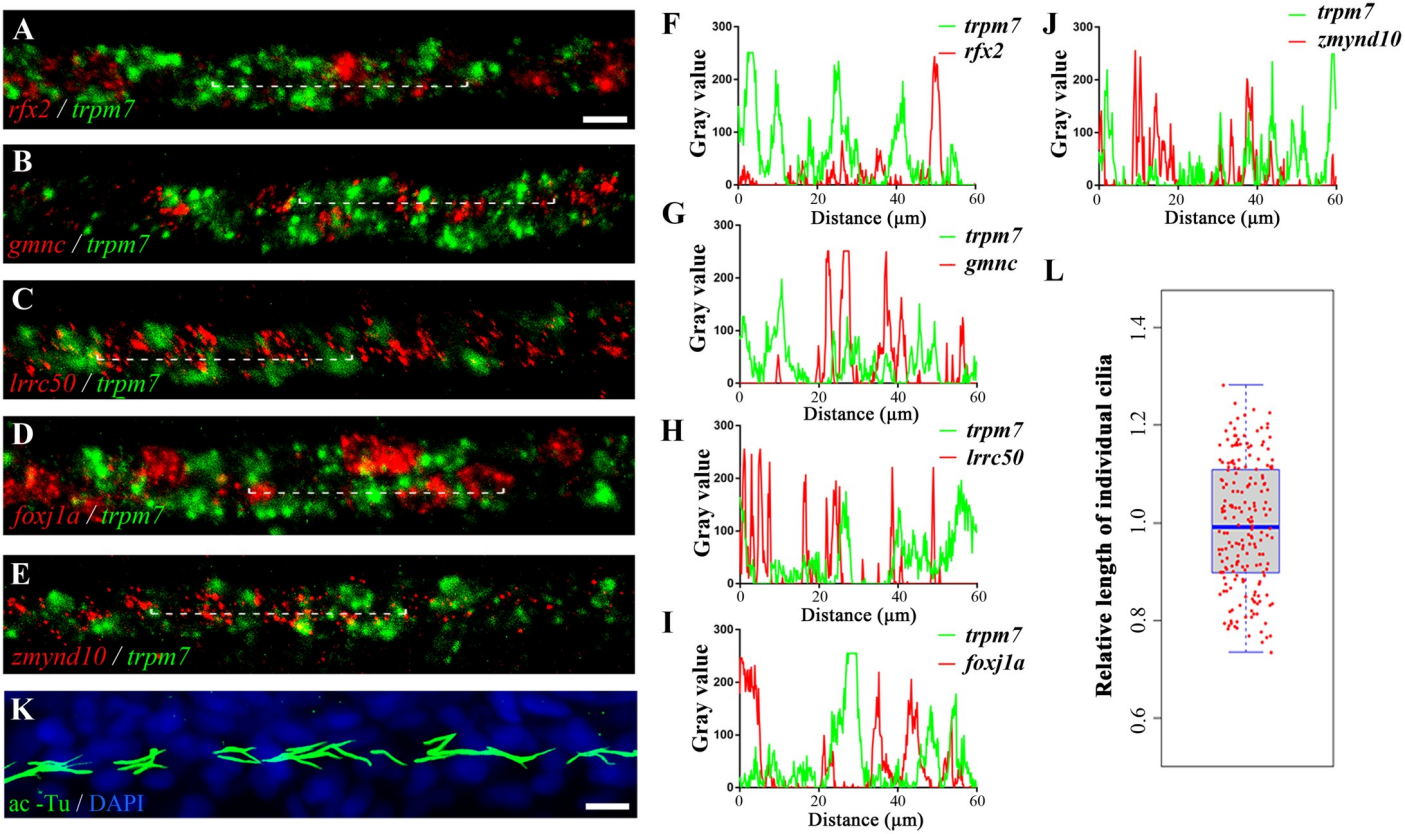
Cilia are not present in the PST principal cells. (A-E) Double fluorescence *in situ* hybridization results showing the expression of *trpm7* and different motile cilia related genes as indicated. (F-J) Line profile plots showing the pixel intensities of green and red channels along the dotted line in panels A-E. (K) Confocal image showing cilia in the PST region of a 24 hpf wild-type larva. Cilia were visualized with anti-acetylated tubulin antibody in green and nuclei were counterstained with DAPI in blue. (L) Box plot graph showing relative length of 198 individual cilia from 33 embryos. Each dot represents a single cilium. Scale bars: 10 μm.

Principal cells have been suggested earlier to form cilia in the pronephric duct region [13, 18]. To clarify from where the extra motile cilia arose in the *e2f4;e2f5* mutants, we further investigated ciliogenesis in the PST region. Using a stable Tg(*β-actin:Arl13b-GFP*) transgene which labels cilia in the pronephric duct, we observed that all the cilia in the PST region of wild-type embryos were motile at 24 hpf (S3 Movie). We further performed double *in situ* hybridization analysis using *trpm7*, a principal cell marker, together with several genes required for ciliary motility, including *rfx2*, *foxj1a*, *zmynd10*, *lrrc50*, as well as *gmnc*, a marker for multiciliated cells. The expression of *trpm7* and all of these motile cilia related genes showed clearly non-overlapping expression pattern (Pearson’s Correlation Coefficient were all close to 0), suggesting that these motile cilia were not differentiated from principal cells (Fig 5A–5J, S6A Fig). Moreover, cilia were of similar length in this region when labeled with anti-acetylated tubulin antibody (Fig 5K and 5L). These findings suggest that contrary to previous reports [13, 18], motile cilia in the PST region do not differentiate on principal cells.

### E2f5 determines principal cell differentiation by regulating *jag2b* expression in MCCs

**Fig 6:**
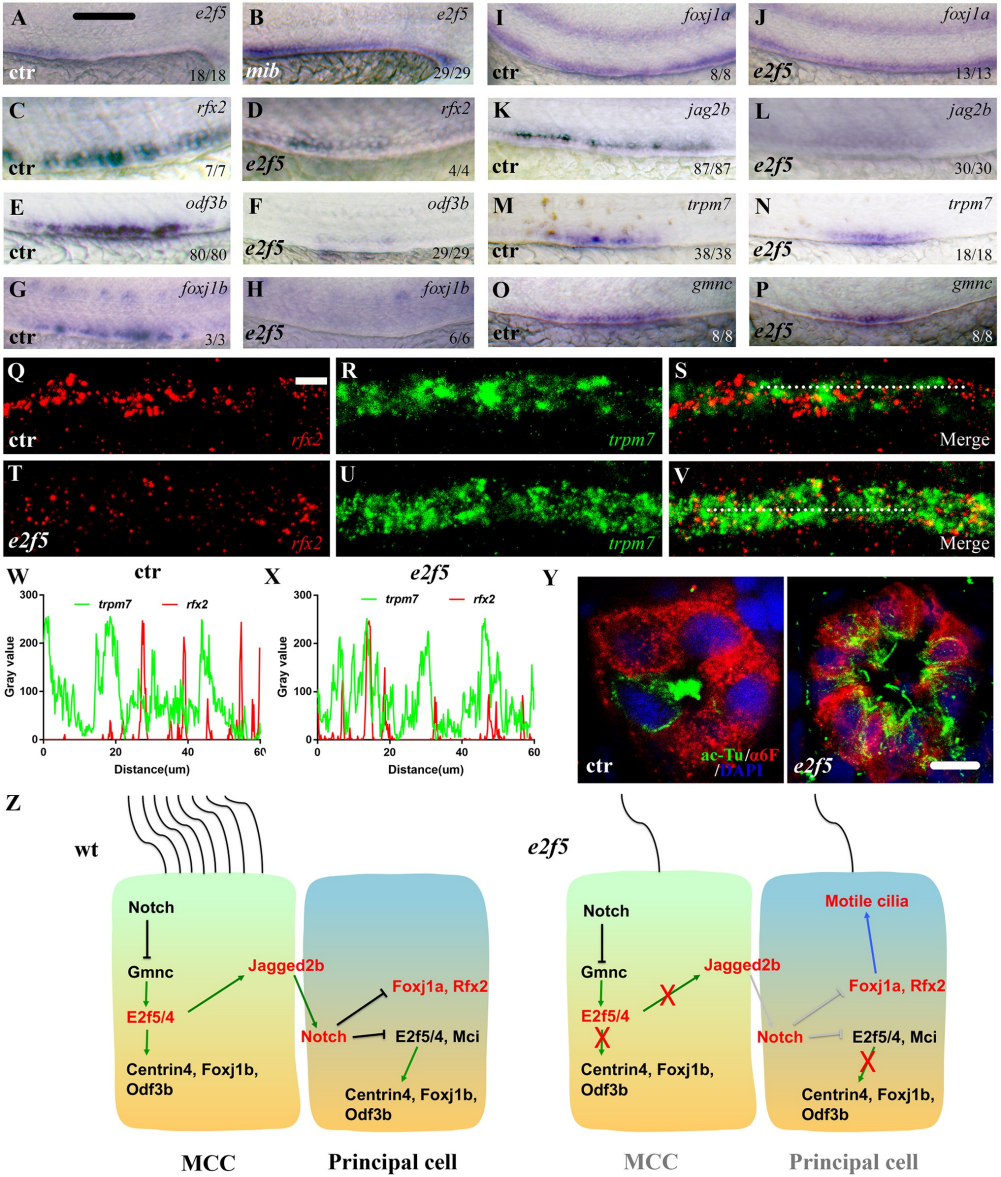
Ectopic motile cilia developed in the principal cells of *e2f5* mutants. (A-P) Whole-mount *in situ* hybridization results showing the expression of *e2f5* and other marker genes in the pronephric duct of 24 hpf control and mutant larvae as indicated. The numbers in the bottom right-hand corners indicate the numbers of embryos with similar staining results (left) and total numbers of embryos analyzed (right). (Q-V) Fluorescence *in situ* hybridization results showing the expression of *rfx2* (red) and *trpm7* (green) in the pronephric tubule of 36 hpf wild-type control (Q-S) and *e2f5* mutant larvae (T-V). (W-X) Line profile plots showing the pixel intensities of green and red channels along the dotted line in panels S and V. (Y) Immunofluorescence results showing the staining of anti-acetylated tubulin (ac-Tu) and α6F antibodies on cross-sections through the pronephric tubules of 5dpf control and *e2f5* mutant larvae. (Z) Model illustrating dual roles of E2f5 during multiciliogenesis. In the absence of E2f5 (maybe also E2f4), both MCC and principal progenitor cells developed a single motile cilium. Scale bars: 500 μm in panels A-P, 10 μm in panels Q-V and 5 μm in panel Y.

As mentioned before, during zebrafish pronephros development, the fate of MCCs and principal cells is determined through the Notch signaling pathway. Zebrafish *mind bomb* (*mib*) mutants are deficient in a ubiquitin ligase essential for Notch signaling, and thus, develop supernumerary MCCs in the pronephric ducts (S7A–S7J Fig) [13]. Consistent with this and the requirement of *e2f5* in MCC formation, the expression of *e2f5* was expanded in *mib* mutants (Fig 6A and 6B). We next examined the expression of a suite of genes associated with MCC development in the *e2f5* mutants to uncover the exact defect in the MCC developmental program. We observed that the expression of *rfx2*, *foxj1b*, *odf3b* and *cetn4*, which are highly expressed in the MCCs, was strongly reduced in *e2f5* mutants, further confirming the defect in multiciliogenesis (Fig 6C–6H, S7K and S7L Fig). On the other hand, the expression of *foxj1a*, a master regulator for motile cilia formation, was maintained in the mutants, suggesting different roles of *foxj1a* and *foxj1b* during MCC differentiation (Fig 6I and 6J).

Unexpectedly, the expression of *jag2b* was also dramatically diminished in *e2f5* mutants (Fig 6K and 6L). Inhibition of Jag2b expression leads to MCC hyperplasia at the expense of principal cells [13, 18]. To test whether the number of MCCs and principal cells changed in *e2f5* mutants, we further stained the mutants with several MCC and principal cells markers. The expression of *trpm7*, together with other markers for transporting epithelial cells were unchanged in the mutants (Fig 6M and 6N, S7M–S7T Fig). Furthermore, the expression of *gmnc*, an early MCC differentiation marker was also unchanged (Fig 6O and 6P). These results suggest that although lateral inhibition was diminished, the number of the progenitor cells of MCCs and principal cells was maintained in the mutants. We hypothesized that extra motile cilia in the PST region of pronephric tubules of the *e2f5* mutants may come from principal cells. To test this, we performed double fluorescence *in situ* hybridization assay with *trpm7* and *rfx2* genes. Rfx2 is a central regulator for ciliogenesis and also a marker for MCCs [18]. In wild-type larvae, *rfx2* and *trpm7* displayed a mutually exclusive expression pattern (Fig 6Q–6S and 6W). By contrast, *rfx2* and *trpm7* transcripts partially colocalized in the mutant pronephric ducts (Fig 6T–6V and 6X). Pearson’s Correlation Coefficient analysis also suggested a positive correlation in the expression of these two genes in the mutants (S6B Fig). Together, these data suggest that some of the principal cells expressed *rfx2* in the mutants. Indeed, double immunostaining with α6F (antibody against chick alpha-1 subunit of the Na^+^/K^+^ ATPase) and acetylated-alpha tubulin antibody showed that cilia developed in the principal cells of *e2f5* mutants, but not in those of wild-type larvae (Fig 6Y).

Together, all of these findings suggest that E2f5, possibly together with E2f4, is required both for multiciliated and principal cell differentiation. In *e2f5* single or *e2f4; e2f5* double mutants, the progenitor cells of MCCs and principal cells developed but failed to further undergo their specific differentiation programs. Instead, both MCCs and principal cells developed single motile cilia (Fig 6Z).

## Discussion

During cell-cycle regulation, E2F5 has been recognized as a transcriptional repressor that inhibits gene expression at G1 phase. In this study, we provide data to show that E2f5 also functions as a transcription activator. In particular, E2f5 activates the transcription of *dmc1* to promote homologous recombination during meiosis. Our data, together with previous reports, suggest that E2f5 also functions as a transactivator to control the expression of genes required for centriole duplication and ciliogenesis during MCC differentiation. Interestingly, when investigating the role of E2f5 through luciferase reporter assay, we found that E2f5 can function as a transcriptional repressor in cultured cells (S8A and S8B Fig). Furthermore, we generated an *e2f5* mutant line carrying the inducible transgene *Tg(fabp10*:*rtTA2s-M2;TRE2*:*EGFP-kras*^*G12V*^*)*, which contains a liver-specific double transgene to induce the expression of EGFP- Kras^G12V^ specifically in the liver that leads to excessive cell proliferation and subsequent carcinogenesis [43, 44]. *e2f5* mutant fish developed significantly enlarged and hyperplastic livers than wild-type siblings, providing corroborating evidence that E2f5 does play an inhibitory role during cell proliferation (S8C and S8D Fig). Thus, our present data together with earlier studies suggest that E2f5 is an ambivalent transcription factor, acting as an activator as well as repressor during development, in a context-dependent manner.

During sexual maturation, juvenile zebrafish usually first develop presumptive ovaries that contain gonocytes, early meiotic oocytes, and perinucleolar oocytes. Later, oocytes in animals that will become females continue to mature. By contrast, those fish with oocyte degeneration and spermatogonia and spermatocyte development develop as males [45]. Whole-mount *in situ* hybridization with *vasa*, a germ cell marker, showed that the number of primordial germ cells (PGCs) appeared to be normal in *e2f5* mutants at early stages. Recovery of females among *e2f5; tp53* double mutants indicates that oocyte apoptosis is the main reason for the loss of females among *e2f5* mutants. These findings also suggest that similar to spermatocyte apoptosis, E2f5 may also regulate homologous recombination during oocyte meiosis. Meiosis arrest in *e2f5* mutants is similar to that reported for *brca2* and *fancl* mutants [35, 36, 46]. Both Brca2 and Fancl function to repair Spo11-induced double strand breaks to initiate meiotic recombination by forming complex with Rad51 and Dmc1 [28]. These data indicate that mutations affecting factors involved in DSB repair will result in meiosis arrest and germ cell apoptosis, which finally causes the male infertility phenotype. We have shown here that E2f5 binds directly to *dmc1* promoter and that the downregulation of *dmc1* expression is the major factor accounting for HR defects in *e2f5* mutants. Although overexpression of Dmc1 increased male fertility in *e2f5* mutants, we failed to recover mutant females, suggesting that other genes regulated by E2f5 also contributes to proper oocyte development.

We have also extended and further clarified the role of E2f5 in the development of the MCCs. Our data are consistent with a model of E2f5 playing cell autonomous and non-cell autonomous functions for the differentiation of the MCCs and the principal cells. *e2f5* expression is regulated by Notch signaling, the major signaling pathway regulating MCC differentiation. E2f5, by interacting with Gmnc, activates the expression of genes required for basal body amplification and multiciliogenesis in the MCCs. This may also include the expression of Jag2b, which activates Notch signaling in the neighboring principal cells and prevents them from adopting the MCC fate (Fig 6Z). In *e2f5* mutants, MCCs failed to form due to the absence of key genes required for centriole duplication. Interestingly, expression of *gmnc*, an early marker for MCCs, is unaffected in *e2f5* mutants (Fig 6O and 6P). Gmnc functions both in the initial step for MCC precursor specification and is also required for later multiciliogenesis [47]. These results suggest that multiciliated progenitor cells develop in the mutants, and the numbers of these cells are unchanged. In the neighboring principal cells, the expression of several ciliary genes is de-repressed due to the absence of inhibitory Notch signaling from the loss of Jag2b in the MCCs. Consequently, the expression of *foxj1a* is activated in these cells, which promotes the formation of a single motile cilium. Interestingly, our data also suggest a functional difference of *foxj1a* and *foxj1b* during multicilia formation. Foxj1b likely contributes to multiciliogenesis in MCCs, while Foxj1a is a general factor for motile cilia differentiation. In *gmnc* mutants, the expression of *foxj1b* is absent while *foxj1a* remains unchanged, which further confirms their functional diversity [19]. Finally, principal cell markers, including *trpm7*, appear to be expressed normally in the *e2f5* mutants. Together, these results suggest that both MCCs and principal cell precursors are specified in the mutants, while multiciliation and lateral inhibition is interrupted due to the loss of E2f5, and this finally results in the formation of single motile cilia in both cell types.

In conclusion, our study shows that besides its function as a transcription repressor during cell division, E2f5 also functions as a transcription activator both in spermatogenesis and multiciliogenesis. This information will help us to further appreciate the genetic complexity in the function of the E2f factors during embryonic and post-embryonic development as well as in adult physiology.

## Materials and methods

### Ethics statement

All zebrafish study was conducted according standard animal guidelines and approved by the Animal Care Committee of Ocean University of China (Animal protocol number: OUC2012316).

### Zebrafish strains and mutants

All zebrafish strains were maintained at 14-hour light / 10-hour dark cycle at 28.5°C. *mib*^*ta52b*^ mutant line was obtained from Dr. Anming Meng. *tp53*^*zdf1*^ mutant line was obtained from Dr. Jianfeng Zhou. The Tg (*fabp10*:*rtTA2s-M2;TRE2*:*EGFP-kras*^*G12V*^) transgenic line was a gift from Dr. Zhiyuan Gong. Zebrafish *e2f4* and *e2f5* mutants were generated with CRISPR/Cas9 system with the following target sequences: *e2f4*: 5’- AGATCTCAAGTTGGAACTGG -3’, *e2f5*: 5’-GGGTGACGAACTTGACTGTG -3’. The single guide RNA and Cas9 mRNA were synthesized using Ambion’s MEGAshortscript T7 Transcription Kit (AM1354). Guide sgRNAs and Cas9 mRNA were co-injected into zebrafish embryos at the one-cell stage.

### Transgenic lines

The transgenic fish expressing *e2f5* or *dmc1* were generated using Multisite Gateway technology. Briefly, the open reading frame of *e2f5* and *dmc1* was amplified from zebrafish cDNA library and cloned into pDONR vector using BP reaction (Invitrogen). For *e2f5* gene, the ORF was cloned into pDONRP2R-P3 vector, then multisite gateway recombination was performed with the following constructs: pDEST vector, p5E-*β-actin*, pME-EGFP and pDONRP2R-P3-*e2f5*. The *dmc1* final construct was generated through multisite gateway with pDEST vector, p5E-*β-actin*, pDONR221-*dmc1* and p3E-IRES-EGFP. Multisite gateway cloning was performed according to the standard protocol from Invitrogen (Life Technologies).

### Whole-mount *in situ* hybridization

Whole-mount *in situ* hybridization was performed according to the standard protocol. The primers used to amplify genes involved in the development of MCCs and principal cells are listed in S1 Table. The *clcnk*, *slc12a1* and *slc4a4a* genes were obtained from Dr. Ying Cao. Double fluorescent *in situ* hybridization was carried out using TSA-plus Fluorescein System (Perkin Elmer) according to manufacturer’s protocol. Images were captured with Leica M165FC microscope or Leica TCS SP8 confocal microscope.

### Immunohistochemical staining, immunofluorescence and TUNEL assay

Zebrafish testes were dissected and fixed in 4% paraformaldehyde (PFA) (w/v) in PBST overnight at 4°C. After gradual dehydration through 30%, 50%, 75%, 95% and 100% ethanol, the testes were embedded in JB4 embedding medium (Polysciences Inc.). Sections and H&E staining were performed as previously described [44]. For immunofluorescence on whole-mount larvae or cryosections through the testis, the following antibodies were used: mouse anti-acetylated tubulin (sigma, T6793), rabbit anti-acetylated tubulin (Cell Signaling Technology, 5335S), anti-glycylated tubulin (EMD, TAP952), anti- γ tubulin (sigma,T5326), anti-γ-H2Ax (Genetex), and a6F (Developmental Studies Hybridoma Bank). TUNEL assay was performed using the *in situ* cell death detection kit (Roche) according to standard protocols from the manufacturer.

### Quantitative PCR

Testes from *e2f5* mutants or control siblings were dissected to isolate total RNA. cDNA was synthesized using PrimeScript 1st strand cDNA Synthesis Kit (Takara). qPCR was performed on the Step One real-time PCR system (Thermo Scientific) using the Eva-Green Master Mix (ABM). The primers used for qPCR are listed in Supplementary S2 Table. Relative gene expression levels were quantified using the comparative Ct method (2^−ΔΔCt^ method) based on Ct values for target genes and zebrafish *β-actin*.

### Chromatin-immunoprecipitation assay

Testes from *Tg*(*β-actin*:*GFP-e2f5*) transgenic fish were first crosslinked in 1.85% PFA for 15 min at room temperature, and subsequently quenched with 0.125M glycine for 5 min with gentle shake, then homogenized with nuclei lysis buffer (50 mM Tris-HCl pH8.0, 10 mM EDTA, 1% SDS, 1 mM PMSF and protease inhibitor APL). The sample was sonicated using Bioruptor sonicator (Diagenode) for 15 min with 30s ON and 30s OFF at high power. 50 μl sonicated samples were saved as input control. Then, 25 μl GFP-Trap-A (Chromotek) was added to the sonicated samples and incubated overnight at 4°C with rotation. After 1 min centrifuge at 1000g, the beads were washed sequentially with wash buffer I (20 mM Tris-HCl pH 8.0, 2 mM EDTA, 1% TritonX-100, 150 mM NaCl and 0.1% SDS), wash buffer II (20 mM Tris-HCl pH 8.0, 2 mM EDTA, 1% TritonX-100, 500 mM NaCl and 0.1% SDS), wash buffer III (10 mM Tris-HCl pH 8.0, 1 mM EDTA, 0.25 M LiCl, 1% NP-40 and 1% Deoxycholate Sodium Salt) and TE buffer (10 mM Tris-HCl pH 8.0 and 1mM EDTA). Finally, 500 μl elution buffer (25 mM Tris-HCl, 10 mM EDTA and 0.5% SDS) were added to elute the DNA-protein complex at 65° for 15 min. The supernatant was transferred to a new tube and 20 μl 5M NaCl was added, then incubated at 65° for 4 hours to reverse crosslinks. Finally, the immunoprecipitated DNA were purified using Phenol/Chloroform/Isoamyl alcohol and further analyzed with regular PCR. The following primers were used for PCR analysis: *dmc1* forward: 5’-GCTGCAAAGCTGAAGTATTC-3’, reverse 5’- CACGTAATTTGGTAACAAG-3’; *rad51* forward 5’- TGCCAGTAGTTTGAATGAGC -3’, reverse 5’- TCACTCACCCGCTAAGCTAC -3’ and *blm* forward 5’- TAGTCCTATTATTAGCGCCG -3’, reverse 5’- AACCAAATAACACAACAAAG -3’.

### Electrophoretic mobility shift assay (EMSA)

The coding region of *e2f5* was amplified from cDNA library and ligated into pDONR221 Gateway entry vector. After LR reaction, full-length coding sequence was further cloned into pET30A vector. The expression of E2f5 was induced by 0.5 mM IPTG and purified with nickel-nitrilotriacetic acid resin column (GE Healthcare). All the synthetic oligonucleotides were FAM-labelled at the 5-terminal (Beijing Genomics Institute). The oligonucleotides were first dissolved in distilled water to a final concentration of 100 μM and annealed with their complementary oligonucleotides to a final concentration of 25 μM. The binding reaction was carried out with 3 μg purified protein, 1μl annealed oligonucleotide in binding buffer (10 mM Tris-HCl pH 8.0, 50 mM NaCl, 1 mM MgCl_2_, 0.5 mM EDTA, 0.5 mM DTT and 4% glycerol). After 40 min incubation at room temperature, the samples were analyzed by 6.5% native polyacrylamide gel electrophoresis and detected using ChemiDoc MP Imaging System (Bio-Rad).

### Chromosome spreading

To determine the stages of meiosis, testes were dissected from wild-type and mutant zebrafish and incubated in 50 μl 1 X PBS. After gently tearing up using a pair of tweezers, 10 μl liquid was transferred into 300 μl hypotonic solution (100 μl 1xPBS plus 200 μl double distilled water) onto an adhesive slide. After 25 min incubation, the cells were fixed in 4% PFA for 5 min, washed three times with PBS, then blocked 30 min with blocking solution (10% goat serum, 3% BSA, 0.05% TritonX-100). The slides were further incubated with Anti-Sycp3 antibody (Abcam, ab150292) for 4–5 hours at room temperature. Then after a brief wash, the secondary antibody was added and the slides were incubated at 37°C for 2 hours. Images were collected with a Leica Sp8 confocal microscope.

### RNA-seq transcriptome analysis

Testes from five wild-type or *e2f5* mutants were dissected and total RNA was isolated using Trizol according to standard protocol. RNA sequencing was performed by Novogene using Illumina HiSeq X Ten (Novogene Bioinformatics Technology Co., Ltd., Tianjin, China). To reduce potential sequencing errors, we repeated this experiment with Annoroad (Annoroad Gene Technology Co., Ltd, Beijing, China). These two sample sets displayed similar changes in gene expression pattern. Both of these RNA-seq data have been deposited to the Sequence Read Archive (SRA) database under accession numbers SRR10854654, SRR10854655, SRR10854656 and SRR10854657 (https://www.ncbi.nlm.nih.gov/bioproject/PRJNA599540). We have mainly shown the data that we obtained from Novogene. For data processing, the reads were mapped to the reference genome index constructed by hisat2 (version 2.1.0) using Danio_rerio.GRCz10.90.gtf and Danio_rerio.GRCz10.dna.chromosome.1.fa. The raw counts of the genes were obtained using featureCounts in the subread version 1.6.2 package. Sample normalization and differential expression analysis was performed using edgeR Bioconductor package. Then, the relative expression level RPKM of the differential genes was calculated according to the traditional formula, and the gene expression heat map was obtained using the pheatmap Bioconductor package.

### High-speed video microscopy

Cilia motility in the PST of the zebrafish pronephric tubules was recorded at 5dpf. Briefly, wild-type or mutant embryos were first treated with 30 μg/ml 1-phenyl 2-thiourea (PTU) to inhibit pigmentation from 24 hpf. At 5dpf, the larvae were anesthetized with 0.01% tricaine, and then placed on top of a cover glass. After removing most of the embryo medium, the cover glass was placed upside down on the center of a depression slide containing 50 μl embryo medium. Cilia movement in the pronephric tubules was recorded with 100 X oil objective on a Leica Sp8 confocal microscope equipped with a high-speed camera (Motion- BLITZ EoSens mini1; Mikrotron, Germany). Cilia movement was captured at rates of 500 frames per second, and playback was set at 25 frames per second.

To record cilia motility in the *Tg(β-actin*:*Arl13b-GFP)* embryos, 24 hpf zebrafish embryos were embedded in the same way as 5dpf larvae and movement of beating cilia was recorded using an Olympus IX83 microscope equipped with a 60X, 1.3 NA objective lens, an EMCCD camera (iXon+ DU-897D-C00-#BV-500; Andor Technology), and a spinning disk confocal scan head (CSU-X1 Spinning Disk Unit; Yokogawa Electric Corporation). Ciliary motility movies were acquired using μManager (https://www.micro-manager.org) at an exposure time of 25 ms, and playback speed of 10 fps. Image processing was performed using ImageJ software (National Institutes of Health, Bethesda, MD, USA).

### Luciferase assay

Partial *dmc1* promoter containing wild-type or mutant E2f5 binding site was cloned into the pGL3 Luciferase Reporter Vector. For the effector vector, full length zebrafish *e2f5* was first cloned into pDONR221vector using BP reaction, then further cloned into pCS2-6xMyc destination vector using LR reaction. We co-transfected the effector and reporter vectors, along with the Renilla luciferase vector, into HEK293 cells using Lipofectamine 2000 (Invitrogen) with standard protocol. Luciferase activity was measured using the Dual Luciferase reporter assay kit (Promega) at 48 hours after transfection.

### Pharmacological treatments

To test the role of E2f5 during cell proliferation *in vivo*, we generated *e2f5* heterozygotes carrying the *Tg(fabp10*:*rtTA2s-M2;TRE2*:*EGFP-kras*^*G12V*^*)* transgene, which drives liver-specific expression of an activating mutation of KRAS to induce excessive cell proliferation after drug treatment. We crossed *e2f5* heterozygotes carrying the EGFP-*kras*^*G12V*^ transgene with *e2f5* heterozygotes, and compared the liver size between *e2f5* homozygotes carrying the EGFP-*kras*^*G12V*^ transgene with wild-type carrying EGFP-*kras*^*G12V*^ transgene. The embryos were treated with 60 μg/mL doxycycline hydrochloride (Sangon Biotech, Shanghai, China) or DMSO starting from 60 hours post fertilization and harvested at 1, 2 and 3 days after treatment. Image capture and statistical analysis were as described previously[44].

### Statistical analysis

Statistical analysis was performed using Microsoft Excel or GraphPad Prism 6 software. All data were presented as mean ±S.D. as indicated in the figure legends. Statistical significance was evaluated by means of the two-tailed Student’s t-test for unpaired data. Pearson Coefficient analysis were performed using the Leica Sp8 confocal colocalization software. A value of p<0.05 was considered statistically significant. All the p values are indicated in the figures.

## Supporting information

S1 Fig Phenotypes of *e2f5* mutants. (A-B) External phenotypes of male and female *e2f5* mutants rescued by *gfp-e2f5* transgene under the regulation of the ubiquitously expressed *β-actin* promoter. (C-D) Dissected testes from wild-type (C) and *e2f5* adult mutant (D). (E-F) External phenotypes of 72 hpf embryos collected from crosses between wild-type male and *e2f5;tp53* double heterozygous female (E) or *e2f5;tp53* homozygous female (F). Scale bars: 1 cm in panel A, B and 1mm in panel C-F.
(TIF)

S2 Fig Expression of *dmc1* was downregulated in *e2f5* mutants. (A) Heat map showing relative expression of genes involved in homologous recombination from RNA-seq transcriptome analysis. The genes are listed according to fold change (FC) and p-value. (B) qPCR results showing the relative expression level of genes involved in homologous recombination in wild-type and *e2f5* mutant testes.
(TIF)

S3 Fig Phenotypes of mature spermatozoa in wild-type and *e2f5* mutants. (A-D) Confocal images showing the phenotypes of mature spermatozoa in wild-type (A) and *e2f5* mutants (B-D). Flagella were labeled with anti-acetylated tubulin antibody in green. Nuclei were stained with DAPI in blue. Scale bar: 5 μm.
(TIF)

S4 Fig Gene ontology (GO) enrichment analysis of differentially expressed genes in the testes of *e2f5* mutants. The genes were clustered according to biological processes. The colors of the bars indicate p adjust value of different GO terms.
(TIF)

S5 Fig Ciliogenesis in *e2f5* and *e2f4;e2f5* double mutants. (A-H) Confocal images showing cilia in the cristae (A-B), spinal canal (SC) (C-D), olfactory pit (OP) (E-F) and PCT of the pronephros (G-H) in 5dpf wild-type and *e2f5* mutants. Cilia were visualized with anti-glycylated tubulin antibodies in green and nuclei were counterstained with DAPI in blue. Arrow in (E) points to cilia bundle of MCCs and asterisk indicates single primary cilia. (I) Diagram showing the genomic structure of *e2f4* locus. The sequences of the wild-type and *e2f4* mutant alleles generated with CRISPR/Cas9 method is shown at the bottom. The sgRNA target sequence and corresponding PAM region are also labeled. (J-M) Confocal images showing the localization of basal bodies visualized with anti-γ tubulin (green) in the olfactory pits of wild-type and mutant larvae as indicated. Arrows point to MCCs characterized by multiple basal bodies. Inserted images are magnified views. Nuclei were stained with DAPI in blue and F-actin was counterstained with phalloidin in red. Scale bars: 10 μm.
(TIF)

S6 Fig Colocalization coefficient analysis by Pearson’s method for genes expressed in MCCs and principal cells. (A) Colocalization analysis of different genes as indicated in 24 hpf wild-type embryos. (B) Colocalization analysis of *rfx2* and *trpm7* expression in the PST of 36 hpf wild-type or *e2f5* mutants as indicated. In panels A and B, each dot represents one zebrafish embryo analyzed.
(TIF)

S7 Fig Expression of pronephric duct marker genes in *mib* and *e2f5* mutants. Whole mount *in situ* hybridization results showing the expression of ciliary genes (A-H, K-L) and marker genes for transporter cells (I-J, M-T) in the pronephric duct of 24 hpf control and mutant embryos as indicated. The numbers of positive/total analyzed embryos are shown in the bottom right-hand corner of each panels.
(TIF)

S8 Fig Zebrafish E2f5 plays repressor role during cell cycle regulation. (A) Diagram showing the constructs used for reporter assays. Part of the promoter region of *dmc1* was used to drive the expression of the luciferase gene. The E2f5 binding site is also indicated. The mutant sequence of E2f5 binding site is the same as used for EMSA assay. (B) Bar graph showing the relative luciferase activity in the different combinations as indicated. Increase in the amount of E2f5 constructs further inhibited luciferase activity. (C) Representative images showing the liver of control and *e2f5* mutants as highlighted by EGFP-Kras^G12V^ expression at different time points after doxycycline treatment. dpt: days post treatment. (D) Dot plot showing the average liver size in wild-type or *e2f5* mutants at different time points after treatment. Scale bar: 200 μm.
(TIF)

S1 Movie High-speed video microscopy showing cilia beating in the pronephric duct of 5dpf wild-type zebrafish larva. (MOV)

S2 Movie High-speed video microscopy showing cilia beating in the pronephric duct of 5dpf *e2f4;e2f5* mutant larva. (MOV)

S3 Movie High-speed video microscopy showing cilia beating in the PST region of pronephric duct in a 24 hpf zebrafish embryo. Cilia were visualized using Tg(*β-actin:Arl13b-GFP)* transgene. Scale bar: 5 μm.
(AVI)

S1 Table Primers used to amplify genes for whole-mount in situ hybridization. (DOCX)

S2 Table Primers used for qPCR analysis. (DOCX)

## References

[1] DeGregori J., Johnson D. G., Distinct and Overlapping Roles for E2F Family Members in Transcription, Proliferation and Apoptosis. *Current molecular medicine* 6, 739–748 (2006). 10.2174/1566524010606070739 17100600

[2] Chen H. Z., Tsai S. Y., Leone G., Emerging roles of E2Fs in cancer: an exit from cell cycle control. *Nature reviews*. *Cancer* 9, 785–797 (2009). 10.1038/nrc2696 19851314 PMC3616489

[3] Dick F. A., Goodrich D. W., Sage J., Dyson N. J., Non-canonical functions of the RB protein in cancer. *Nature reviews*. *Cancer* 18, 442–451 (2018). 10.1038/s41568-018-0008-5 PMC6693677 29692417

[4] Ertosun M. G., Hapil F. Z., Osman Nidai O., E2F1 transcription factor and its impact on growth factor and cytokine signaling. *Cytokine & growth factor reviews* 31, 17–25 (2016). 26947516 10.1016/j.cytogfr.2016.02.001

[5] Attwooll C., Lazzerini Denchi E., Helin K., The E2F family: specific functions and overlapping interests. *The EMBO journal* 23, 4709–4716 (2004). 10.1038/sj.emboj.7600481 15538380 PMC535093

[6] Humbert P. O. et al, E2F4 is essential for normal erythrocyte maturation and neonatal viability. *Molecular cell* 6, 281–291 (2000). 10.1016/s1097-2765(00)00029-0 10983976

[7] Rempel R. E. et al, Loss of E2F4 activity leads to abnormal development of multiple cellular lineages. *Molecular cell* 6, 293–306 (2000). 10.1016/s1097-2765(00)00030-7 10983977

[8] Ruzhynsky V. A. et al, Cell cycle regulator E2F4 is essential for the development of the ventral telencephalon. *The Journal of neuroscience*: *the official journal of the Society for Neuroscience* 27, 5926–5935 (2007). 17537963 10.1523/JNEUROSCI.1538-07.2007 PMC6672261

[9] Danielian P. S., Hess R. A., Lees J. A., E2f4 and E2f5 are essential for the development of the male reproductive system. *Cell cycle* 15, 250–260 (2016). 10.1080/15384101.2015.1121350 26825228 PMC4825840

[10] Lindeman G. J. et al, A specific, nonproliferative role for E2F-5 in choroid plexus function revealed by gene targeting. *Genes & development* 12, 1092–1098 (1998). 9553039 10.1101/gad.12.8.1092 PMC316727

[11] Spassky N., Meunier A., The development and functions of multiciliated epithelia. *Nature reviews*. *Molecular cell biology* 18, 423–436 (2017). 10.1038/nrm.2017.21 28400610

[12] Brooks E. R., Wallingford J. B., Multiciliated cells. *Current biology*: *CB* 24, R973–982 (2014). 10.1016/j.cub.2014.08.047 25291643 PMC4441396

[13] Liu Y., Pathak N., Kramer-Zucker A., Drummond I. A., Notch signaling controls the differentiation of transporting epithelia and multiciliated cells in the zebrafish pronephros. *Development* 134, 1111–1122 (2007). 10.1242/dev.02806 17287248

[14] Mori M. et al, Notch3-Jagged signaling controls the pool of undifferentiated airway progenitors. *Development* 142, 258–267 (2015). 10.1242/dev.116855 25564622 PMC4302835

[15] Tsao P. N. et al, Notch signaling controls the balance of ciliated and secretory cell fates in developing airways. *Development* 136, 2297–2307 (2009). 10.1242/dev.034884 19502490 PMC2729343

[16] Marcet B. et al, Control of vertebrate multiciliogenesis by miR-449 through direct repression of the Delta/Notch pathway. *Nature cell biology* 13, 693–699 (2011). 10.1038/ncb2241 21602795

[17] Deblandre G. A., Wettstein D. A., Koyano-Nakagawa N., Kintner C., A two-step mechanism generates the spacing pattern of the ciliated cells in the skin of Xenopus embryos. *Development* 126, 4715–4728 (1999). 10518489 10.1242/dev.126.21.4715

[18] Ma M., Jiang Y. J., Jagged2a-notch signaling mediates cell fate choice in the zebrafish pronephric duct. *PLoS genetics* 3, e18 (2007). 10.1371/journal.pgen.0030018 17257056 PMC1781496

[19] Zhou F. et al, Gmnc Is a Master Regulator of the Multiciliated Cell Differentiation Program. *Current biology*: *CB* 25, 3267–3273 (2015). 10.1016/j.cub.2015.10.062 26778655

[20] Terre B. et al, GEMC1 is a critical regulator of multiciliated cell differentiation. *The EMBO journal* 35, 942–960 (2016). 10.15252/embj.201592821 26933123 PMC5207319

[21] Arbi M. et al, GemC1 controls multiciliogenesis in the airway epithelium. *EMBO reports* 17, 400–413 (2016). 10.15252/embr.201540882 26882546 PMC4772991

[22] Stubbs J. L., Vladar E. K., Axelrod J. D., Kintner C., Multicilin promotes centriole assembly and ciliogenesis during multiciliate cell differentiation. *Nature cell biology* 14, 140–147 (2012). 10.1038/ncb2406 22231168 PMC3329891

[23] Chong Y. L., Zhang Y., Zhou F., Roy S., Distinct requirements of E2f4 versus E2f5 activity for multiciliated cell development in the zebrafish embryo. *Developmental biology* 443, 165–172 (2018). 10.1016/j.ydbio.2018.09.013 30218642

[24] Ma L., Quigley I., Omran H., Kintner C., Multicilin drives centriole biogenesis via E2f proteins. *Genes & development* 28, 1461–1471 (2014). 24934224 10.1101/gad.243832.114 PMC4083089

[25] Mori M. et al, Cytoplasmic E2f4 forms organizing centres for initiation of centriole amplification during multiciliogenesis. *Nature communications* 8, 15857 (2017). 10.1038/ncomms15857 28675157 PMC5500891

[26] Zhao H. et al, The Cep63 paralogue Deup1 enables massive de novo centriole biogenesis for vertebrate multiciliogenesis. *Nature cell biology* 15, 1434–1444 (2013). 10.1038/ncb2880 24240477

[27] Wang Y., Copenhaver G. P., Meiotic Recombination: Mixing It Up in Plants. *Annu Rev Plant Biol* 69, 577–609 (2018). 10.1146/annurev-arplant-042817-040431 29489392

[28] Neale M. J., Keeney S., Clarifying the mechanics of DNA strand exchange in meiotic recombination. *Nature* 442, 153–158 (2006). 10.1038/nature04885 16838012 PMC5607947

[29] Sehorn M. G., Sigurdsson S., Bussen W., Unger V. M., Sung P., Human meiotic recombinase Dmc1 promotes ATP-dependent homologous DNA strand exchange. *Nature* 429, 433–437 (2004). 10.1038/nature02563 15164066

[30] Cloud V., Chan Y. L., Grubb J., Budke B., Bishop D. K., Rad51 is an accessory factor for Dmc1-mediated joint molecule formation during meiosis. *Science* 337, 1222–1225 (2012). 10.1126/science.1219379 22955832 PMC4056682

[31] Da Ines O. et al, Meiotic recombination in Arabidopsis is catalysed by DMC1, with RAD51 playing a supporting role. *PLoS genetics* 9, e1003787 (2013). 10.1371/journal.pgen.1003787 24086145 PMC3784562

[32] Bisig C. G. et al, Synaptonemal complex components persist at centromeres and are required for homologous centromere pairing in mouse spermatocytes. *PLoS genetics* 8, e1002701 (2012). 10.1371/journal.pgen.1002701 22761579 PMC3386160

[33] Saito K., Siegfried K. R., Nusslein-Volhard C., Sakai N., Isolation and cytogenetic characterization of zebrafish meiotic prophase I mutants. *Dev Dyn* 240, 1779–1792 (2011). 10.1002/dvdy.22661 21594953

[34] Mahadevaiah S. K. et al, Recombinational DNA double-strand breaks in mice precede synapsis. *Nat Genet* 27, 271–276 (2001). 10.1038/85830 11242108

[35] Rodriguez-Mari A. et al, Roles of brca2 (fancd1) in oocyte nuclear architecture, gametogenesis, gonad tumors, and genome stability in zebrafish. *PLoS genetics* 7, e1001357 (2011). 10.1371/journal.pgen.1001357 21483806 PMC3069109

[36] Shive H. R. et al, brca2 in zebrafish ovarian development, spermatogenesis, and tumorigenesis. *Proc Natl Acad Sci U S A* 107, 19350–19355 (2010). 10.1073/pnas.1011630107 20974951 PMC2984219

[37] Zhao W., Wiese C., Kwon Y., Hromas R., Sung P., The BRCA Tumor Suppressor Network in Chromosome Damage Repair by Homologous Recombination. *Annu Rev Biochem* 88, 221–245 (2019). 10.1146/annurev-biochem-013118-111058 30917004 PMC7004434

[38] Sun Y., McCorvie T. J., Yates L. A., Zhang X., Structural basis of homologous recombination. *Cell Mol Life Sci* 77, 3–18 (2020). 10.1007/s00018-019-03365-1 31748913 PMC6957567

[39] Kagawa W., Kurumizaka H., From meiosis to postmeiotic events: uncovering the molecular roles of the meiosis-specific recombinase Dmc1. *FEBS J* 277, 590–598 (2010). 10.1111/j.1742-4658.2009.07503.x 20015079

[40] Chen J. et al, Disruption of dmc1 Produces Abnormal Sperm in Medaka (Oryzias latipes). *Sci Rep* 6, 30912 (2016). 10.1038/srep30912 27480068 PMC4969596

[41] Drummond I. A., Davidson A. J., Zebrafish kidney development. *Methods Cell Biol* 100, 233–260 (2010). 10.1016/B978-0-12-384892-5.00009-8 21111220

[42] Zhao C., Malicki J., Genetic defects of pronephric cilia in zebrafish. *Mech Dev* 124, 605–616 (2007). 10.1016/j.mod.2007.04.004 17576052

[43] Nguyen A. T. et al, An inducible kras(V12) transgenic zebrafish model for liver tumorigenesis and chemical drug screening. *Disease models & mechanisms* 5, 63–72 (2012). 21903676 10.1242/dmm.008367 PMC3255544

[44] Kang Y., Xie H., Zhao C., Ankrd45 Is a Novel Ankyrin Repeat Protein Required for Cell Proliferation. *Genes (Basel)* 10 (2019). 10.3390/genes10060462 PMC6628321 31208154

[45] Kossack M. E., Draper B. W., Genetic regulation of sex determination and maintenance in zebrafish (Danio rerio). *Curr Top Dev Biol* 134, 119–149 (2019). 10.1016/bs.ctdb.2019.02.004 30999973 PMC6894417

[46] Rodriguez-Mari A. et al, Sex reversal in zebrafish fancl mutants is caused by Tp53-mediated germ cell apoptosis. *PLoS genetics* 6, e1001034 (2010). 10.1371/journal.pgen.1001034 20661450 PMC2908690

[47] Lu H. et al, Mcidas mutant mice reveal a two-step process for the specification and differentiation of multiciliated cells in mammals. *Development* 146 (2019). 10.1242/dev.172643 30877126

