# Supplementary figures and images for: E2f5 is a versatile transcriptional activator required for spermatogenesis and multiciliated cell differentiation in zebrafish
Source: PLoS Genet. 2020 Mar 20;16(3):e1008655. doi: 10.1371/journal.pgen.1008655 (PMC7112233; doi:10.1371/journal.pgen.1008655)

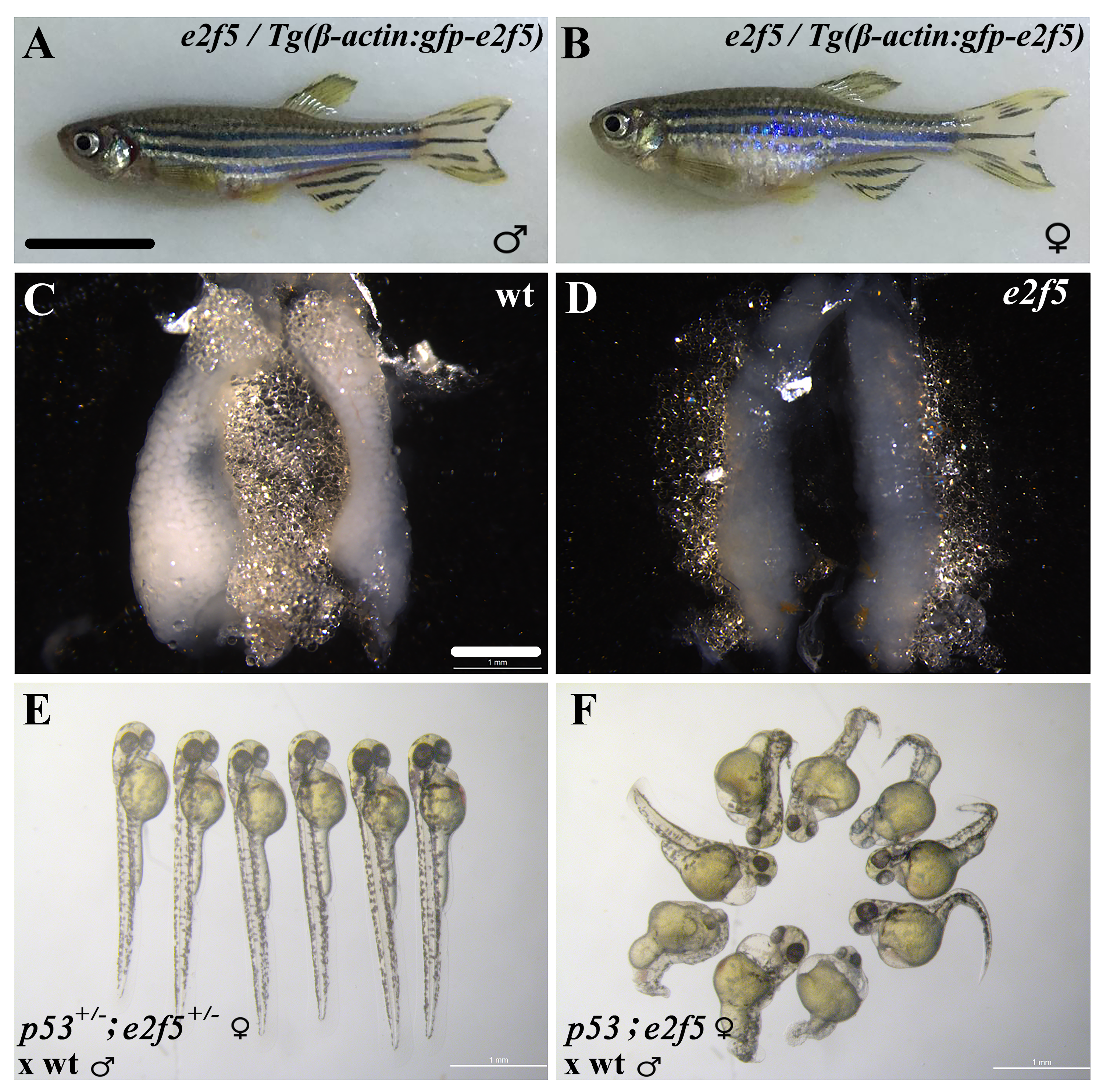

Supplement: S1 Fig — (A-B) External phenotypes of male and female e2f5 mutants rescued by gfp-e2f5 transgene under the regulation of the ubiquitously expressed β-actin promoter. (C-D) Dissected testes from wild-type (C) and e2f5 adult mutant (D). (E-F) External phenotypes of 72 hpf embryos collected from crosses between wild-type male and e2f5;tp53 double heterozygous female (E) or e2f5;tp53 homozygous female (F). Scale bars: 1 cm in panel A, B and 1mm in panel C-F. (TIF) [file pgen.1008655.s001.tif]

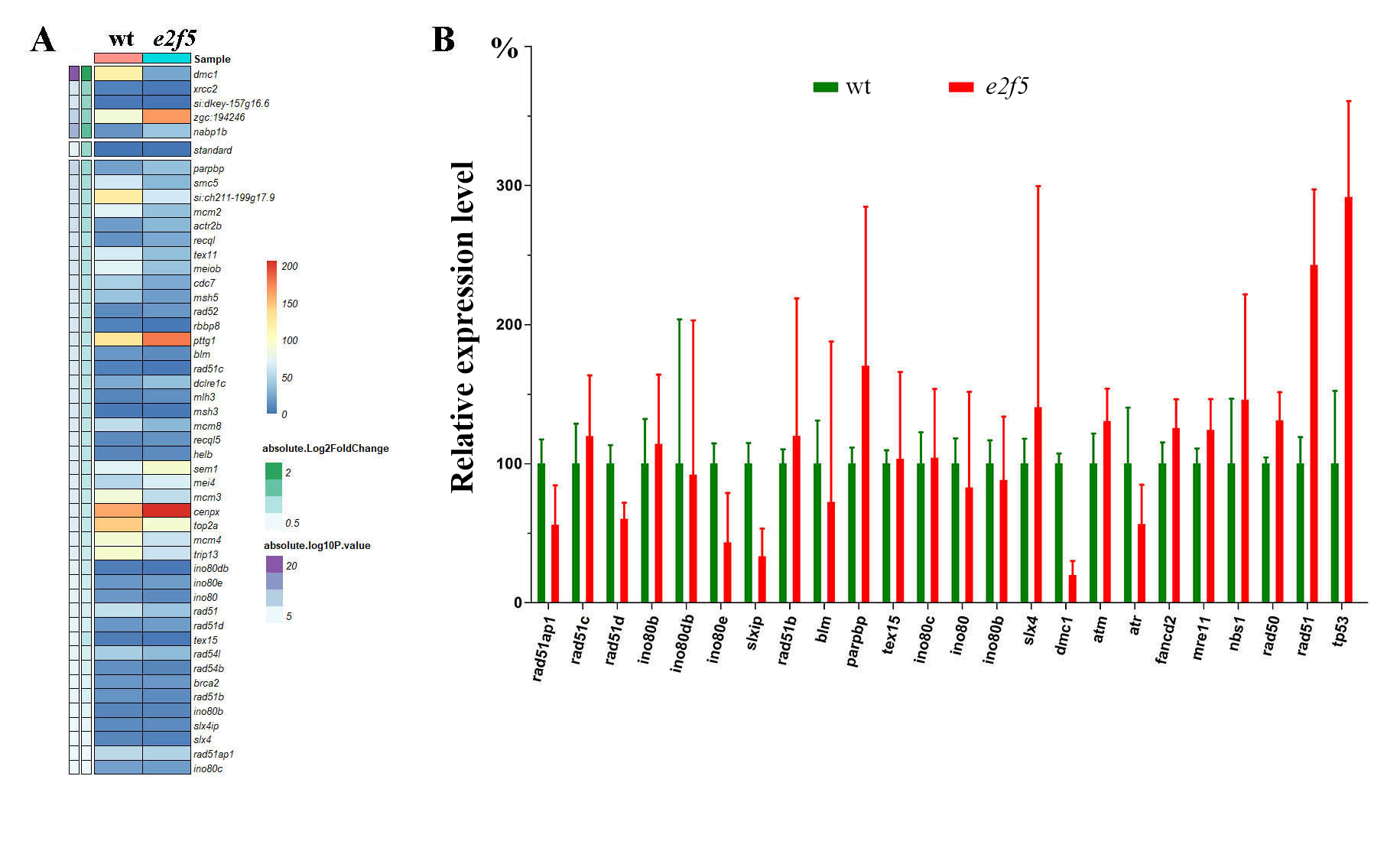

Supplement: S2 Fig — (A) Heat map showing relative expression of genes involved in homologous recombination from RNA-seq transcriptome analysis. The genes are listed according to fold change (FC) and p-value. (B) qPCR results showing the relative expression level of genes involved in homologous recombination in wild-type and e2f5 mutant testes. (TIF) [file pgen.1008655.s002.tif]

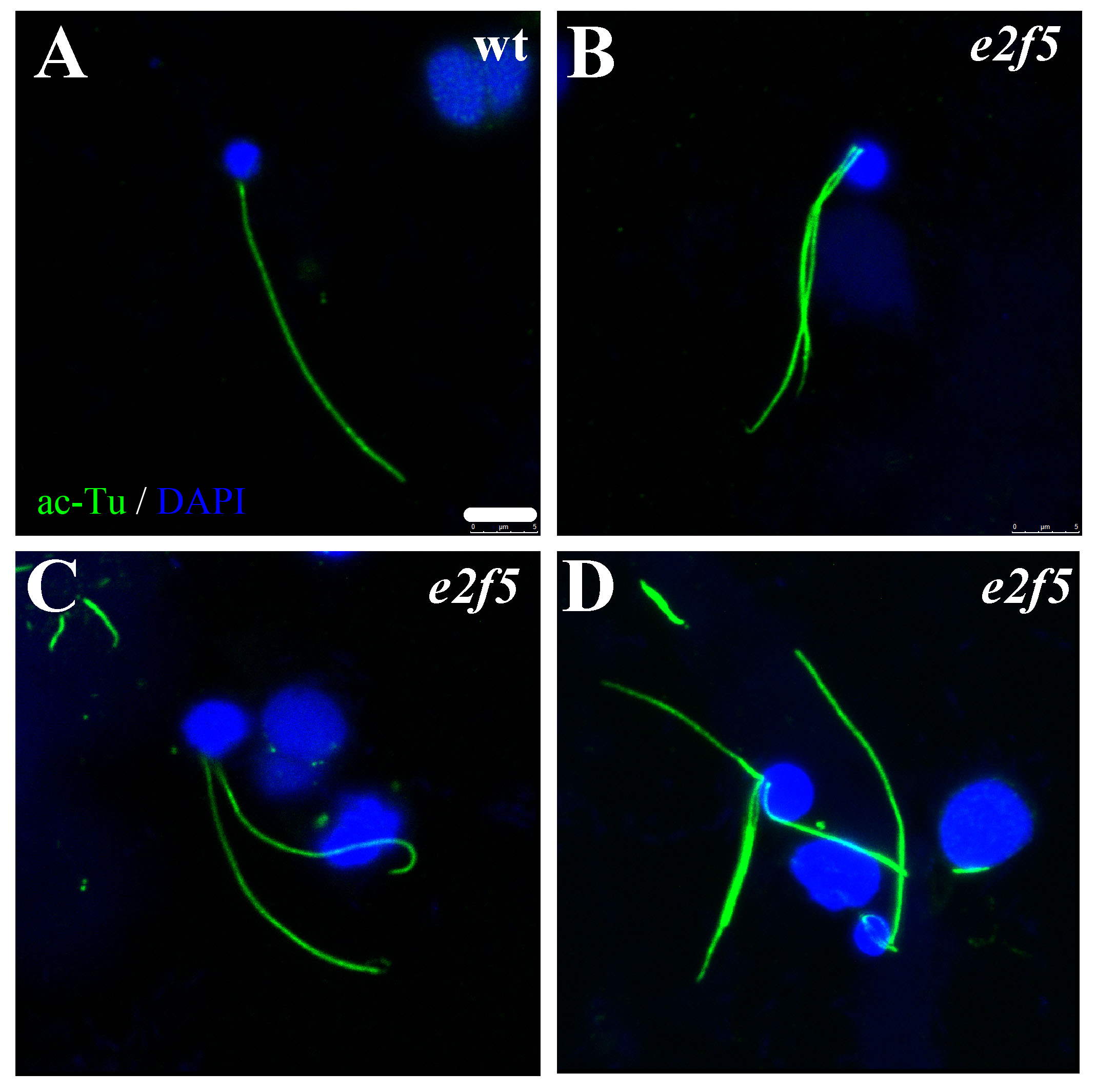

Supplement: S3 Fig — (A-D) Confocal images showing the phenotypes of mature spermatozoa in wild-type (A) and e2f5 mutants (B-D). Flagella were labeled with anti-acetylated tubulin antibody in green. Nuclei were stained with DAPI in blue. Scale bar: 5 μm. (TIF) [file pgen.1008655.s003.tif]

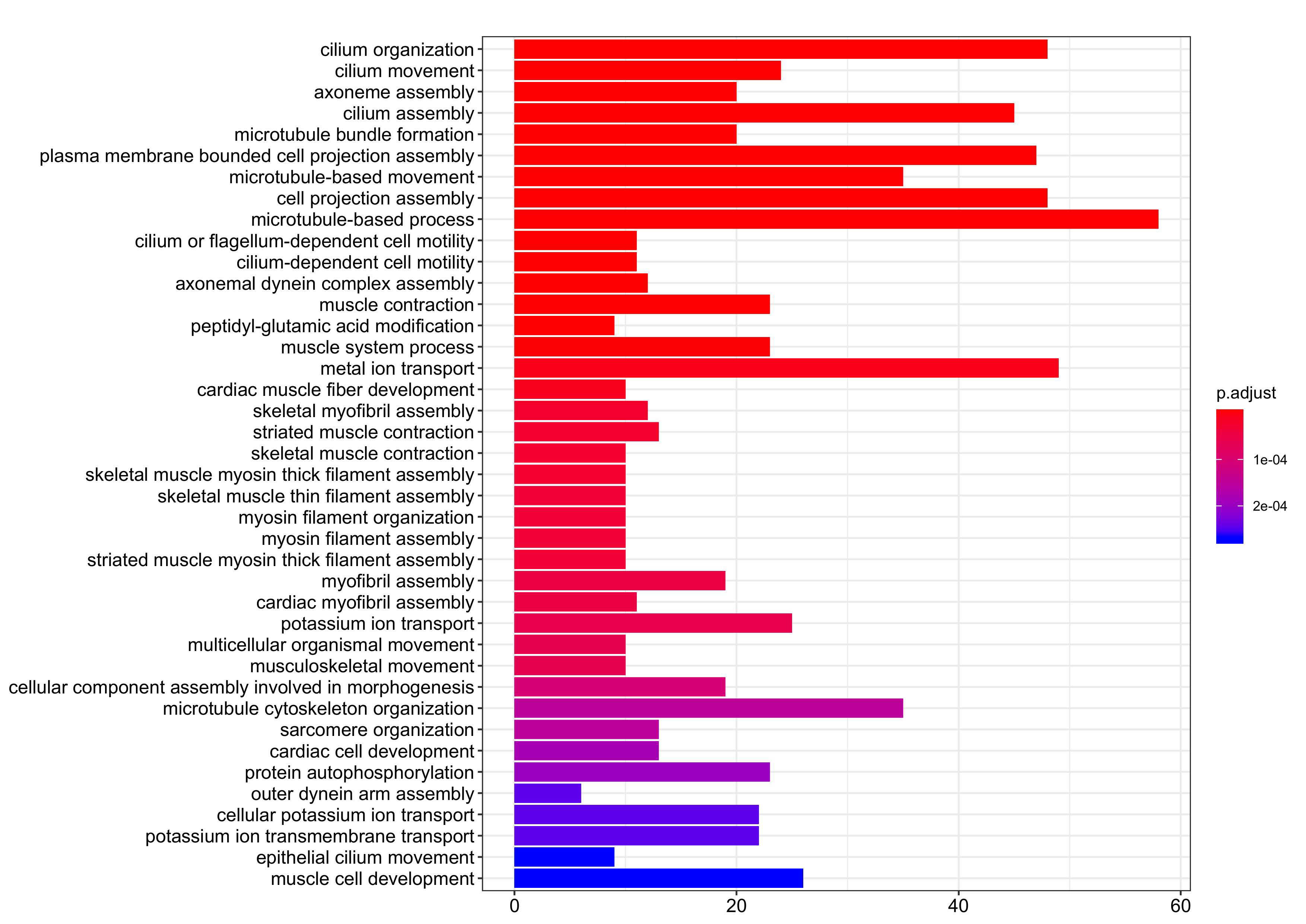

Supplement: S4 Fig — The genes were clustered according to biological processes. The colors of the bars indicate p adjust value of different GO terms. (TIF) [file pgen.1008655.s004.tif]

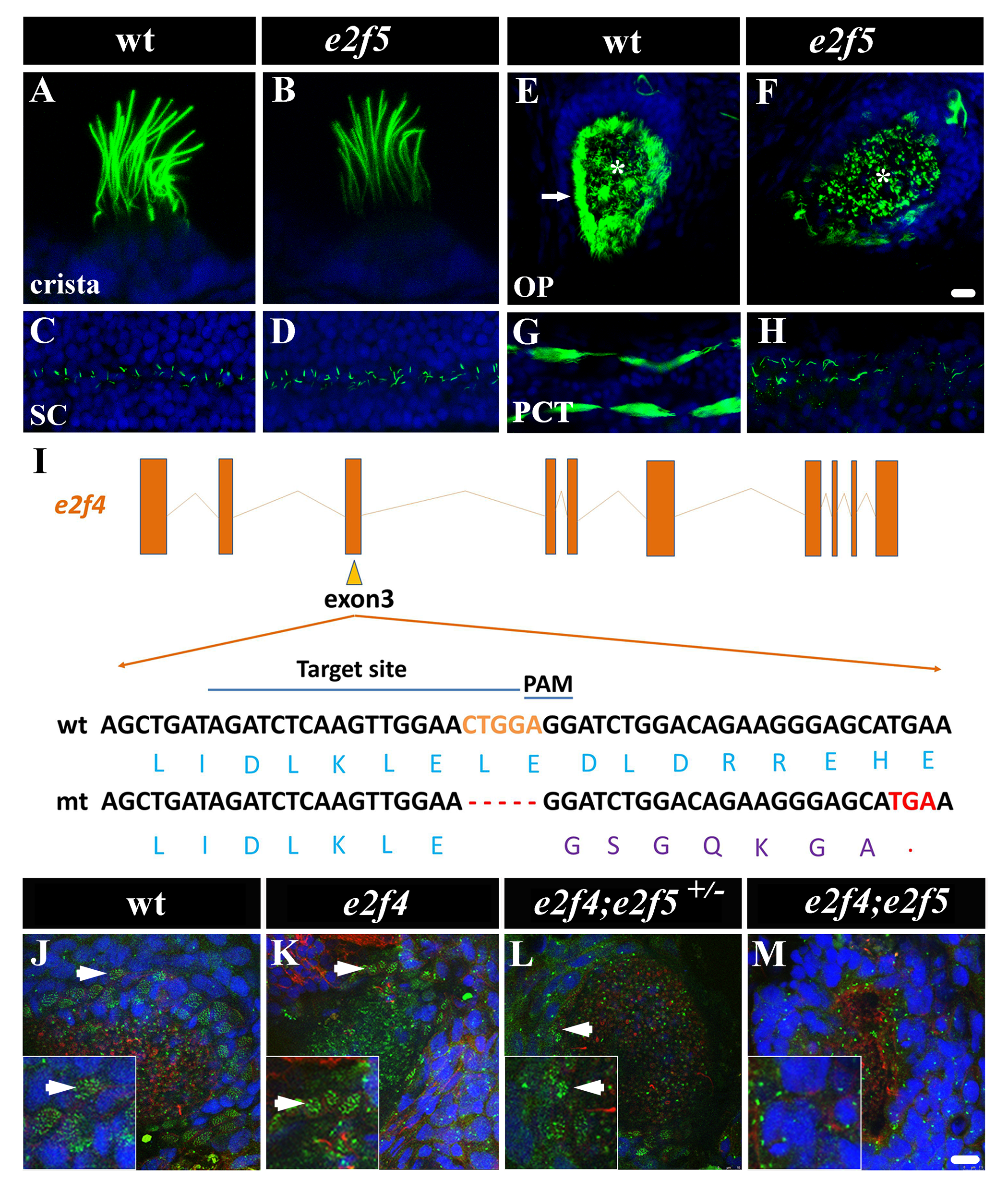

Supplement: S5 Fig — (A-H) Confocal images showing cilia in the cristae (A-B), spinal canal (SC) (C-D), olfactory pit (OP) (E-F) and PCT of the pronephros (G-H) in 5dpf wild-type and e2f5 mutants. Cilia were visualized with anti-glycylated tubulin antibodies in green and nuclei were counterstained with DAPI in blue. Arrow in (E) points to cilia bundle of MCCs and asterisk indicates single primary cilia. (I) Diagram showing the genomic structure of e2f4 locus. The sequences of the wild-type and e2f4 mutant alleles generated with CRISPR/Cas9 method is shown at the bottom. The sgRNA target sequence and corresponding PAM region are also labeled. (J-M) Confocal images showing the localization of basal bodies visualized with anti-γ tubulin (green) in the olfactory pits of wild-type and mutant larvae as indicated. Arrows point to MCCs characterized by multiple basal bodies. Inserted images are magnified views. Nuclei were stained with DAPI in blue and F-actin was counterstained with phalloidin in red. Scale bars: 10 μm. (TIF) [file pgen.1008655.s005.tif]

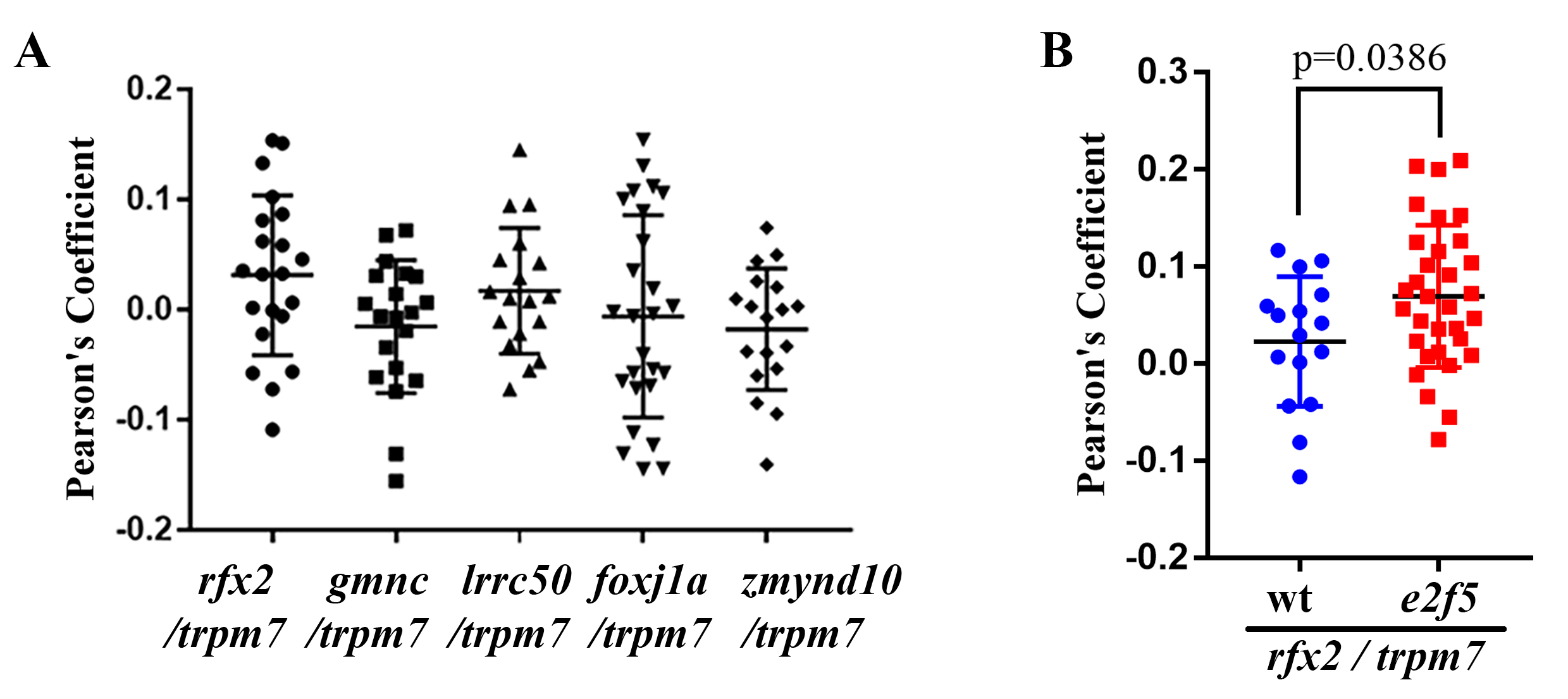

Supplement: S6 Fig — (A) Colocalization analysis of different genes as indicated in 24 hpf wild-type embryos. (B) Colocalization analysis of rfx2 and trpm7 expression in the PST of 36 hpf wild-type or e2f5 mutants as indicated. In panels A and B, each dot represents one zebrafish embryo analyzed. (TIF) [file pgen.1008655.s006.tif]

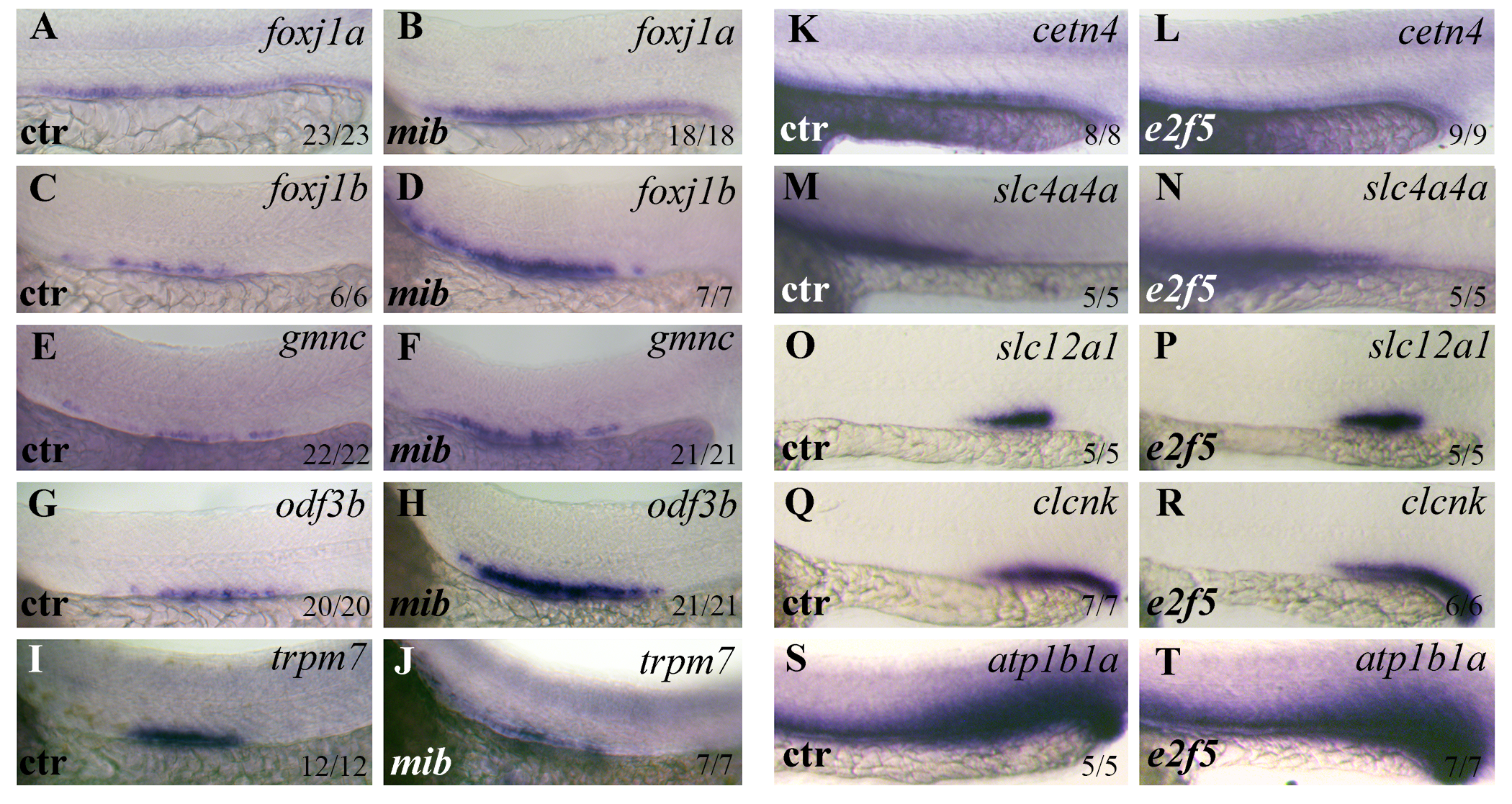

Supplement: S7 Fig — Whole mount in situ hybridization results showing the expression of ciliary genes (A-H, K-L) and marker genes for transporter cells (I-J, M-T) in the pronephric duct of 24 hpf control and mutant embryos as indicated. The numbers of positive/total analyzed embryos are shown in the bottom right-hand corner of each panels. (TIF) [file pgen.1008655.s007.tif]

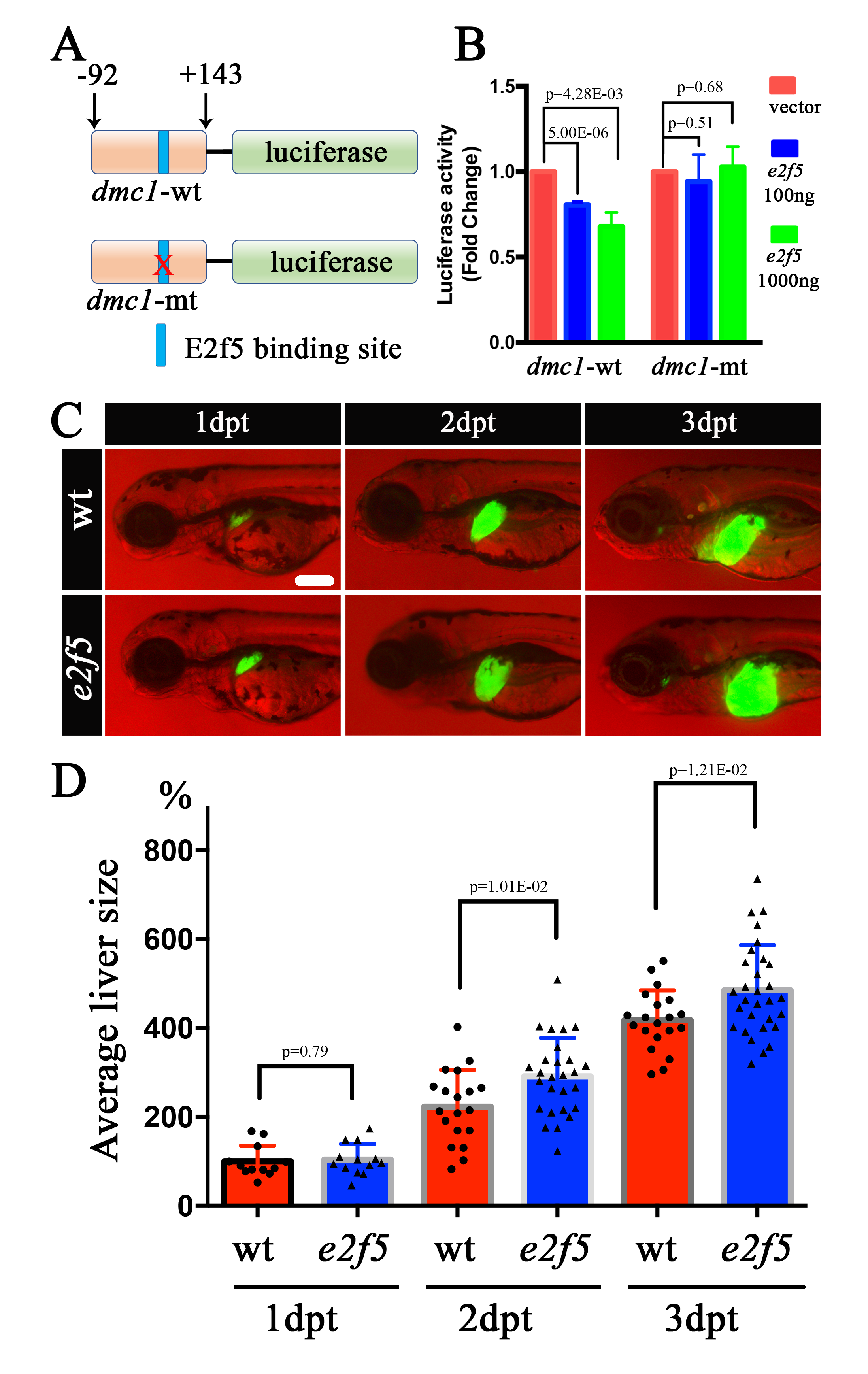

Supplement: S8 Fig — (A) Diagram showing the constructs used for reporter assays. Part of the promoter region of dmc1 was used to drive the expression of the luciferase gene. The E2f5 binding site is also indicated. The mutant sequence of E2f5 binding site is the same as used for EMSA assay. (B) Bar graph showing the relative luciferase activity in the different combinations as indicated. Increase in the amount of E2f5 constructs further inhibited luciferase activity. (C) Representative images showing the liver of control and e2f5 mutants as highlighted by EGFP-KrasG12V expression at different time points after doxycycline treatment. dpt: days post treatment. (D) Dot plot showing the average liver size in wild-type or e2f5 mutants at different time points after treatment. Scale bar: 200 μm. (TIF) [file pgen.1008655.s008.tif]
