# Supplementary material for: E2f5 is a versatile transcriptional activator required for spermatogenesis and multiciliated cell differentiation in zebrafish
Source: PLoS Genet. 2020 Mar 20;16(3):e1008655. doi: 10.1371/journal.pgen.1008655 (PMC7112233; doi:10.1371/journal.pgen.1008655)
Supplement: S1 Table — (DOCX) [file pgen.1008655.s012.docx]

**S1 Table Primers for MCCs and principal cells**

| gene | forward | reverse |
| --- | --- | --- |
| *atp1b1a* | AAGGAAGGGGACAACATCC | ATACTGTCGTTGGATGCTGG |
| *cetn4*  *foxj1a*  *foxj1b*  *gmnc*  *jagge2b*  *lrrc50*  *rfx2*  *odf3b*  *trpm7*  *zmynd10* | CTCACGCAGGTTACAACACG  CCGACAATTTAGACGACAGC  ACCCGCAACACCTCTTTTAC  CTGTCACCTCAACTTCAACG  TCATCCCTTTTCACTTCGCC  GAAGGAACAAGAACTGAGGC  AGCATTACCATTCCCAGCAG  ACAATCGGCGCAATAATAAC  TATCAAACGCTGCTGAATCC  TGCTGGTGATTGTGTTCTTG | TCAGGCGTTTACAGCTTCAG  TGGAGAGCTGGATTGATCTG  TCGTCCTCGTTGCTATAACC  GTGAAGTGTTGAGCGGTTG  CAAGGCTTCCCCATACACTC  CCATTAGATTTAACACCCGC  TTGCCATGTACTGCGTGTCC  GCTTAATCTTCACCATCCAC  TGATGAAGTGGTTGTGTCTG  TTCGTCACTTGGTATTTGGC |
