# Supplementary material for: E2f5 is a versatile transcriptional activator required for spermatogenesis and multiciliated cell differentiation in zebrafish
Source: PLoS Genet. 2020 Mar 20;16(3):e1008655. doi: 10.1371/journal.pgen.1008655 (PMC7112233; doi:10.1371/journal.pgen.1008655)
Supplement: S2 Table — (DOCX) [file pgen.1008655.s013.docx]

| **S2 Table Homologous repair qPCR primers** | | |
| --- | --- | --- |
| gene | forward | reverse |
| *atm* | TGTGGAGGTGTTGCTGTATG TGCTGAATGAGGAGGTTTAC | |
| *atr* | ATAAACCCAGCGTGCCTTTC AGAATCGCTCCACCACCACT | |
| *fancd2* | CGAAAAAGTCCCAGATTCCC TTCGGTCCAGCCACTTTAAC | |
| *mre11a* | GCCATTAGAGGGAACGACAC AGTTGGGTCATCATGGTTGC | |
| *nbs1* | CGAATCTGGAGGGGAAAGTG CCCTTCATTATCCACACACG | |
| *rad50* | TCTGGGAGTGAGGAGTTTTG CAGCCACAGCATCTCCATTC | |
| *rad51* | CTGAACGGTATGGTCTGGTG GGGATCTGCTGAAAACATGG | |
| *rad52* | ATGGATTATAGCAGCGGGAG AAAGCACTGACTCCAACGTA | |
| *rad51ap1*  *rad51b*  *rad51c*  *rad51d*  *dmc1*  *parpbp*  *slx4*  *slx4ip*  *tex15*  *ino80db*  *ino80*  *ino80b*  *ino80c*  *ino80da*  *ino80e*  *blm*  *tp53* | GATTTTGCCTGTGTGAAACC TCCGTCTGACTTGGTTGTAG  ATCTTTCGAGGAGGATTCTG GCTTTCTGGGTTCATGTTCT  CTAGATGATGCTATTGGCGG CCACATTAAGTTCCTCCAGA  CTTTGTCTCATGGAATCCGG TGTCAGCTCAGTTATTTCCC  GGCCGACATAAAGAAACTGA CATACTCTCCACACCTCCTC  CCTCATCCGAAAAGAGTACG CCTCCTGACAACTCTCTGTA  CTGATATGTGGGACGATTGG GTGGGTGTATTGATGGATCT  AGTTCAAGCAGACAAAGAGG CTGCTACTTGAAATTGGCCT  AGCATAAAGTCTAGCAAGGAG AGAGTGTATTTCGGAGTGAG  TAGTGTCATATCAGTGCCAG GAGGTCACTTGATGTTGTTC  CTATGGAAGGAAGGCAACAC CTCTTTGCAGTTCTTCTGTG  TGACGATGAAGACTCTGATG TTCTTCAGCTCTCCGTTATC  CGGAGTATCAAAAGCAAGGG AGCAGTCTCTACATCAACAG  CAAGTACAACAGTCAGCGAT CCGAGTCCTCCTCATAAAAG  GCAGAAGTAGAGGTGGATTA CCATCACTGTCTGAGGAAAC  TGATGACGCTCTCTTTAGTG GCCAGTTTTGACACCTTCTC  GAAGAACAGCCTCAGCCATC GTTATCTCCATCCGGGGTTC | |
